# Mapping employment dynamics in public agencies with payroll data: A methodological framework with an application to Chile

**DOI:** 10.1371/journal.pone.0316386

**Published:** 2024-12-31

**Authors:** Mauricio Herrera, Daniel Brieba

**Affiliations:** 1 Faculty of Engineering, Universidad del Desarrollo, Santiago, Chile; 2 School of Government, Universidad Adolfo Ibáñez, Santiago, Chile; University of Amsterdam, NETHERLANDS, KINGDOM OF THE

## Abstract

This study introduces a novel, replicable methodology for analyzing employment dynamics within public sector agencies, focusing on turnover and staff longevity. The methodology is designed to be generalizable and applicable to diverse national contexts where detailed administrative data is available. Using payroll data from over 325,000 Chilean civil servants (2006—2020), we apply mixed-effects Cox survival models and linear mixed models to examine patterns of employment stability across state agencies. By incorporating Propensity Score Matching, we further enhance the causal interpretation of turnover changes, especially in post-election years. Finally, we introduce two key metrics—Service Frailty and Relative Turnover Difference—to quantify long-term stability and short-term, post-electoral disruptions. Our findings highlight substantial differences in turnover patterns between regular and post-election years, as well as significant inter-agency heterogeneity in turnover and employee longevity, largely driven by latent agency characteristics. While major covariates like contract type and staff rank account for some variation, much of the disparity stems from agency-specific factors. This framework offers precise, cross-nationally comparable benchmarks for understanding public sector employment dynamics. Additionally, the methodology contributes to the literature by providing transparent and scalable tools for analyzing workforce stability across different contexts.

## 1 Introduction

In political science and public administration, there has long been an interest in understanding the role of state bureaucracies in addressing governance challenges, such as inefficiency, corruption, and poor policy outcomes. A significant body of research emphasizes that meritocratic, politically insulated, and professionalized bureaucracies are key to improving governance and achieving better policy results [1–6]. Employment stability is particularly important for the effective functioning of state agencies, as stable staff enable the development of expertise, increase corporate coherence, and foster long-term horizons [7–10]. In contrast, high turnover rates can disrupt an agency’s ability to fulfill its mission and negatively impact policy continuity.

Despite the common tendency to treat “the state” as a unitary actor, its ministries, departments, and agencies (“agencies” for short) often differ significantly in terms of quality [11–13]. The “pockets of effectiveness” literature highlights how some agencies are more insulated from political pressures, allowing them to maintain higher levels of professionalization and stability, while others are more exposed to patronage practices [8, 14–16]. Such variation suggests that agency-level heterogeneity within a state is a critical factor in understanding overall state performance.

Until fairly recently, the study of bureaucracies was limited by the lack of detailed data on individual bureaucratic careers. However, the increasing availability of large surveys (e.g. [17]) and individual-level payroll data offers a new level of scope and precision for analyzing bureaucratic employment dynamics. Specifically, many new studies have exploited payroll data to causally link political shocks such as elections and their outcomes to employment (e.g. [18–21]). However, with some major exceptions [8, 22], few studies have exploited such data to study variation between agencies within a single state. This study contributes to both these literatures by developing a methodological approach that uses payroll data to map employment dynamics across state agencies. By examining patterns of agency turnover and staff longevity, we aim to map the extent of agency heterogeneity both over time and in response to political transitions, which are key moments when patronage practices are most likely to manifest.

We apply this methodology to Chile’s civil service, pursuing four goals: (1) to characterize the levels and trends of staff turnover in the state as a whole and in individual agencies, both in regular and post-electoral years; (2) to analyze the extent to which these differences in turnover can be explained by major agency- and individual-level covariates, as opposed to agency-specific effects; (3) to explore whether the observed differences between regular and post-electoral year turnover can be considered causal, using Propensity Score Matching (PSM) [23] and related techniques to suggest causality; and (4) to develop quantifiable indicators that rank and map agencies based on their long-term and short-term (post-electoral) staffing dynamics.

We use data from over 325,000 Chilean bureaucrats spanning 15 years (2006–2020). We apply mixed linear models to assess how turnover patterns differ across agencies and how these patterns evolved over this period, particularly focusing on how turnover rates vary between regular years and periods of political transition. We introduce an overall measure of how much turnover variance is driven by within-agency versus between-agency differences, and how this proportion compares between regular and post-electoral years. This metric is highly comparable across countries. In addition, we use mixed-effects Cox survival models to analyze staff longevity over our full time period. These models allow us to capture the effects of both individual- and agency-level characteristics on employee longevity while accounting for agency-specific random effects that may explain unobservable characteristics (such as patronage) that produce agency heterogeneity. We also introduce key indicators to quantify service stability over the short and long run, and which are derived from these models.

A key advantage of this method is its transparency and replicability. Our method relies primarily on payroll data, and specifically on employee tenure within an agency, using this single metric to map agency heterogeneity and produce easily interpretable results, such as turnover rates. In contrast to approaches like those of Bersch K. P. et al. in [24], which require multiple variables like wage levels or party membership to map agency heterogeneity through abstract concepts such as capacity and autonomy, our method is less data-intensive and more replicable across different contexts. By focusing on tenure, we offer results that can be directly compared across countries without the need for multidimensional and sometimes country-idiosyncratic data.

Our application of this methodology to Chile’s central government employment data over a 15-year period yields several important findings. First, turnover at the state level remained relatively stable despite three government changes that produced consecutive alternations in power between the two main (center-left and center-right) party coalitions. Second, we observe a significant spike in turnover during post-electoral years, suggesting that political transitions exert considerable pressure on employment stability, and that this relationship is likely to be causal. Third, there are substantial differences in turnover across agencies, with particularly large variations between agencies in post-election years; in such years, almost two thirds of all turnover variance is due to between-agency rather than to within-agency variation. Finally, we find that major agency-level and individual-level covariates explain only a small portion of the overall variance, indicating that agency-specific effects play a significant role in shaping employment dynamics. These findings suggest that patronage practices—which are not directly observable—may be a key factor contributing to agency heterogeneity.

In sum, this paper makes two main contributions. First, it offers a parsimonious and replicable quantitative methodology for assessing staff turnover and longevity that serves as “proof of concept” for other countries where similar payroll data becomes available. Our focus on individual agencies, rather than aggregating all state employment data, provides a more granular understanding of bureaucratic dynamics. Though some prior studies have examined individual-level turnover [25] and others have explored variation in agency capacity and autonomy within a single state [8], this is, to our knowledge, the first study to characterize and model variation in staff turnover dynamics at the agency level. Second, we offer an empirical characterization of turnover dynamics within Chile’s civil service, contributing new insights and detailed information that can serve as a benchmark for other cases in Latin America and beyond. This research is relevant for the comparative study of bureaucratic dynamics and for broader discussions about “islands of excellence” and state heterogeneity.

The remainder of this paper is structured as follows. Section 2 discusses the theoretical underpinnings of civil service employment stability and agency heterogeneity. Section 3 discusses the data and outlines the models used in our analysis, while Section 4 presents the findings. Section 5 discusses the results and their substantive implications. Finally, Section 6 discusses the implications of our methodological contribution, its limitations, and future avenues for research.

## 2 Theoretical framework

A hallmark of Weberian bureaucracies is their foundation on long-term civil service careers, which promote integrity and rule-abiding conduct [1, 4, 26]. Career officials have been found to outperform political appointees [27]. A stable civil service is also more trustworthy for the private sector, shielding long-term investments and projects from morally hazardous behavior by elected officials [7]. By contrast, elevated turnover rates can shorten bureaucrats’ time horizons, reduce mutual accountability and “esprit de corps”, lower accumulated expertise, and lead to policy instability, [2, 7, 9, 28]. Turnover thus affects both agency capacity—the degree to which an agency has the resources to fulfill its mission, such as “esprit de corps” and a qualified workforce [1, 25, 26]—and agency autonomy—the independence from political principals [8, 26]. Agencies with high turnover also incur costs related to vacancies and the need to recruit and train new employees. More generally, ample evidence indicates that short tenure and high rotation levels are detrimental to organizational performance in both the public and private sectors [29–35].

This research speaks to two strands of the literature that have dealt with issues of bureaucratic stability and turnover. The first of these has focused on studying patronage –the discretionary hiring of bureaucrats by politicians [36]—and electoral cycles. Most of this literature has focused on the more developed civil services of Western Europe and North America. Given their more robust employment protections, this bureaucratic politicization seems to occur only at the top of the hierarchy—at the position of agency heads, top management positions, and the like. The finding that turnover at this level is in fact influenced by electoral cycles, despite civil service rules ensuring employee protection, has been reported for Sweden [37], Germany [38], the UK [39], South Korea [40], and the US [25], among others. Even in the latter case, which as a presidential system is usually regarded as being more politicized at the top than Western European parliamentary systems, Bolton et al. [25] report that rotation in top-level managers increases after an election only from about 7% to 9.3%—a jump of 23% over a non-electoral year. In countries with less developed civil service systems, politicization of bureaucratic positions is generally thought to be greater, according to cross-national measures based on expert surveys [41]. In countries with weaker or non-existent civil service systems, patronage can percolate down the entire hierarchy, as occurs in some Latin American countries [42]. In contexts with high scarcity of advanced human capital, patronage can even be concentrated at the bottom, as Brierley [18] shows occurs in Ghana. This literature, however, is mainly concerned with establishing the extent and patterns of politicized turnover, and is mostly focused on turnover at the top of bureaucratic hierarchies. As such, it treats the state as a whole, ignoring agency variation, and given its focus on causal identification, is generally not concerned with measuring turnover in non-transition years (an exception is Bolton et al. [25]).

The second strand—and closest to this work—has focused on agency variation within national states. States are not monolithic; agencies within the same state can vary widely in their levels of professionalism and insulation from political pressures [8, 43, 44]. Indeed, “within-country, cross-agency diversity in capacity often overwhelms the variation encountered across public sectors considered in their entireties” [11]. Thus, inferring the capacity or autonomy of the state as a whole from the behavior of one or a few agencies is risky, particularly when the relative ranking of performance across sectors or agencies may vary between countries, leading to what has been called the “levels of analysis” problem for studying bureaucracies cross-nationally [11]. Again, an important reason for agency variation is rooted in the politics of patronage. Motivations for patronage include inducing policy alignment in the bureaucracy [36, 45], securing electoral support by providing jobs to loyalists, who may perform electoral services in return [46], and building a legislative majority in Congress by offering patronage to partisan allies [47]. Only the first of these reasons is frequent in more developed countries. In contexts where civil services are less institutionalized, all three motivations can result in a trade-off between hiring competent staff and rewarding political allies. In such contexts, heads of government may adopt a dual strategy: protecting key agencies (e.g., central banks, finance ministries) while leaving other agencies open to patronage, leading to a heterogeneous state where some parts function as “islands of excellence” or “pockets of effectiveness” while others are subject to political pressures [12, 14, 16, 44].

While these two strands of literature have advanced our understanding of bureaucratic employment dynamics, the ability to systematically map their variation at the agency level has been hampered by limited data. Payroll data is particularly well-suited to this task, as it offers direct, objective information on employment patterns rather than relying on perceptions. Its scope and granularity also allows for the analysis of agency dynamics and their variation. Closest to our study, the pioneering work of Bersch et al. [8] used payroll data to build factor scores for capacity and autonomy in agencies of the Brazilian federal government, finding significant variation across both dimensions. Their data, like ours, is “*intra-national, focusing on individual bureaucracies within the national state; objective, relying on administrative data concerning individual actors rather than subjective assessments by experts; and generated independently of outcomes*” [8]. While valuable, their method relies on factor analysis that incorporates multiple variables (e.g., employment longevity, wage levels, proportions of expert staff, partisanship) to operationalize abstract concepts like capacity and autonomy, making it data-intensive, less replicable across contexts, and harder to interpret. For instance, the category of “expert career” staff is highly idiosyncratic to Brazil (see [24] for details), while partisanship data for public servants is protected or unavailable in many other countries. These factors hinder replication in other contexts and the comparability of findings.

In contrast, this study helps address this gap by developing a replicable methodology for the study of agency heterogeneity and employment dynamics across the state using mixed linear and survival models. This methodological framework provides a standardized and transparent way to measure levels and trends in turnover, as well as mapping differences in agency stability, offering a scalable solution that can be directly applied and compared across different national contexts.

Importantly, from a theoretical standpoint, employment stability affects both capacity and autonomy (as discussed above). We therefore analyze turnover both in “regular” years and in post-election years. The latter are key moments when patronage practices are most likely to manifest. As new governments come into power, they may seek to replace bureaucratic staff with politically aligned personnel, particularly in agencies that are more exposed to political pressures. This approach allows us to distinguish empirically between short-term turnover, which reflects political transitions and affects agency autonomy, and long-term employment dynamics, which impact an agency’s capacity. For the latter, we go beyond the usual focus on the literature with turnover (post-electoral or otherwise) and model employee longevity using survival models, which better capture these longer-term dynamics and the impact of both individual-level and agency-level determinants on employment tenure. We develop indicators that capture and map agency heterogeneity through standardized indicators along these two (short-term and long-term) dimensions.

We apply this method to data from the Chilean central government, basing our measures on individual-level data of civil servants’ careers over 15 years and 77 agencies for which there is full, continuous data from January 2006 to April 2020. Since individuals are nested within agencies, and these are nested within ministries, we can construct indicators for higher levels based on individual data. Empirically, our models allow us to answer the following questions regarding bureaucratic dynamics, as applied to the Chilean state:

1. How does agency turnover vary, both during regular years and post-electoral years, and what are the trends in turnover over time?
2. To what extent can differences in turnover and employee longevity be explained by observable agency- and individual-level covariates, versus latent agency-specific factors?
3. How do electoral shocks affect turnover patterns, and can these effects be identified as causal?
4. Which agencies are more exposed to political shocks, which are more stable in regular years, and how do these two dynamics relate to each other?

To answer the first two questions, we estimate agency turnover using mixed models [48] and study individual employment longevity using Cox survival models [49]. These models account for both individual- and agency-level covariates, enabling us to assess the degree to which observable factors explain turnover patterns, as opposed to latent agency effects. Our approach provides a rigorous, empirical framework for understanding civil service stability, heterogeneity, and its evolution over time. To explore whether the observed differences in turnover can be attributed to political transitions rather than coincidental factors, we employ Propensity Score Matching (PSM) [23] and mediation analysis [50]. These methods allow us to better isolate the impact of electoral shocks on employment dynamics, offering a more robust understanding of whether political transitions cause the observed turnover patterns. Finally, our study offers a standardized, replicable framework for cross-national comparisons by developing quantifiable indicators that map agencies according to their long-term employee longevity and short-term turnover patterns, offering a structured way to rank and map agency heterogeneity in different national contexts. *Service Frailty*, derived from the Cox model, captures latent, long-term organizational or political factors that shape employee survival in a given agency, while *Relative Turnover Difference* measures the short-term disruption in staff turnover caused by electoral cycles. Overall, our empirical findings and measures contribute to the broader comparative research agenda in public administration.

### 2.1 Case study: Context and data

Chile is one of the most politically stable countries in South America. Its bureaucracy is likewise considered to be one of the most well-developed in the region, along with Brazil’s [51]. The Quality of Government cross-country expert survey of bureaucracies shows Chile’s bureaucracy to be fairly meritocratic, ranking 20 out of 86 countries in the sample. However, in the survey question pertaining specifically to patronage, Chile ranks closer to the middle of the rankings [41]. Thus, Chile is an interesting case to study because its intermediate position is likely more representative of turnover and patronage dynamics across the world than that of more developed countries that typically occupy the top positions in these comparisons.

Since Chile’s return to democracy in 1990 and until 2010, the country was governed by a stable center–left multiparty coalition. During this time, the party system was largely stable, and presidential transitions occurred regularly. In March 2010 power was transferred to a center-right coalition. This inaugurated a more competitive scenario since power returned to the center-left (with an enlarged coalition) in March 2014, but in turn, the center-right returned to power in March 2018. Thus, since our data begins in January 2006 and ends in April 2020, the data covers four changes of government, including three which were transitions from one political coalition to their rivals.

We curated publicly available staffing data from Chilean State agencies web scraped from publicly available Transparency portals mandated by Chilean law. The data preparation process presented substantial challenges, entailing the consolidation of information from over 200 agencies across 24 ministries, encompassing regional and provincial delegations, regional housing services, and all line ministries and their respective agencies (except the health sector). The data spans over 15 years (2006—2020) in a monthly structure. The final dataset contains over 15.7 million monthly observations of over 325,000 individual civil servants. Ensuring data utility required disambiguating public servant identification to be able to track each individual over time, which was achieved with a high degree of accuracy. Processes for data intake, homologation, cleaning, and disambiguation were streamlined through automation using Amazon Web Service cloud infrastructure. Detailed information on the data scraped from the Transparency Portal, as well as the pre-processed datasets used in this analysis, is available in the supporting information S1 File. The curated datasets for this study can be downloaded from [52].

The resulting data structure has several levels, with individuals nested within agencies and agencies nested within ministries. The longitudinal nature of the data enables a quantitative analysis of civil servant careers and agency evolution over time, facilitating a comprehensive examination of national bureaucracies. Using this data, agencies can be delineated by various quantitative indicators, including staff longevity and turnover.

Importantly, individuals can be hired under three modalities in the state: a permanent contract, a yearly renewable contract, and a temporary contract. Though only the permanent contract awards full civil service protection, the yearly contract is virtually as stable and people under this modality have equal legal responsibilities and status as individuals under permanent contracts. For instance, they also have a grade and a rank, which together determine their salary. On the other hand, temporary contracts mix two situations: people who are hired for genuinely temporary tasks that may last anything from a few weeks to a few months, to people who are hired for regular tasks under the guise of a temporary contract for budgetary reasons. Overall, this contract modality is substantially more unstable. Brieba et al. [53] also suggest, through a descriptive analysis of the data, that employees’ rank may also help explain employment stability, with higher ranks—the managerial and professional—having more job instability than lower ranks, such as administrative or technical positions. Therefore, we will account for both these factors—contract modality and job rank—when studying turnover and longevity.

## 3 Materials and methods

### 3.1 Linear mixed models for staff turnover

Staff turnover refers to the proportion of employees departing from an agency within a given year. It is calculated by dividing the number of departing public servants by the total staff count within the agency for that year.

**Fig 1:**
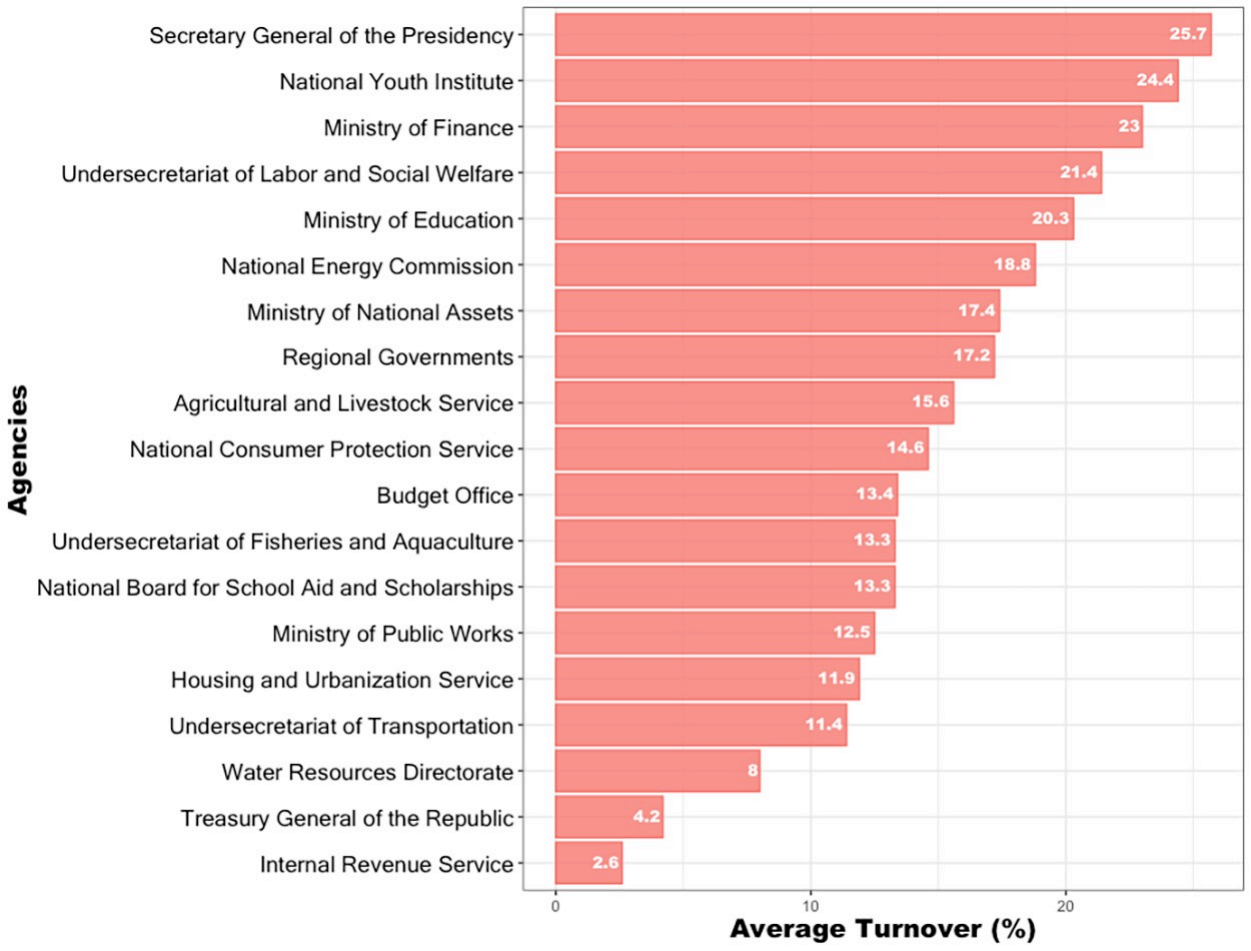
Average staff turnover (percentage) for a random sample of twenty State agencies. The figure shows wide variability in the levels of staff turnover between state agencies, from “stable” agencies to others showing very high levels of staff turnover. (Source: authors).

Fig 1 illustrates the average staff turnover for 20 agencies taken from 2006 to April 2020. The graph shows wide variability in the levels of staff turnover between agencies, highlighting stable agencies with low staff turnover, such as the *Treasury General of the Republic* and the *Internal Revenue Service*, among others. In contrast, other agencies such as the *Secretary General of the Presidency* and *National Youth Institute*, among others, stand out because of their instability, showing very high levels of staff turnover.

**Fig 2:**
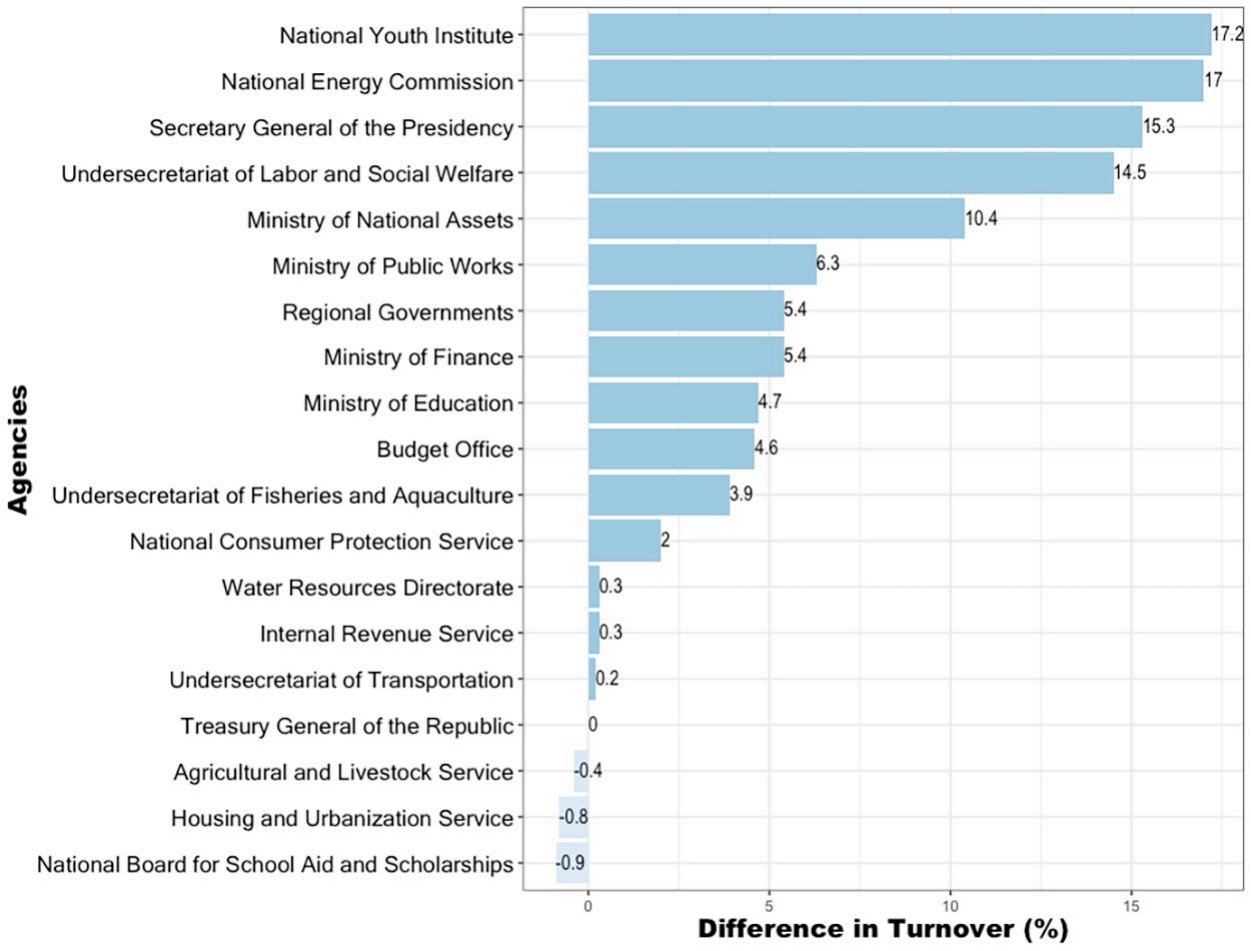
Difference between the mean staff turnover in the first year of a new administration and the base or regular turnover in non-administration (regular) years for a sample of twenty agencies. The difference in agency staff turnover measured in the first year of a new administration compared to the base staff turnover measured in non-administration (regular) years is a proxy for the independence of State agencies concerning political cycles. (Source: authors).

Examining variation in agency staff turnover between the first year of a new administration and regular years offers insight into the degree of independence of state agencies from political–electoral cycles. As depicted in Fig 2, which illustrates the difference in average staff turnover during the first year of a new administration and regular years across twenty state agencies during the monitoring period (2006–2020), large disparities appear. Certain agencies, such as the *National Youth Institute* and *National Energy Commission* exhibit substantial discrepancies, indicative of heightened susceptibility to political cycles.

Figs 1 and 2 thus suggest a significant degree of turnover heterogeneity between agencies, both overall and in post-electoral years, which in the following section is modeled formally using Linear Mixed-Effects Models [48].

#### The unconditional mean model

This model aims to compare how much variation in turnover in the data is due to differences between agencies and how much of it is due to variation within agencies. We calculate this model separately for the first year of new administrations and the remaining years.

The motivation for this model is as follows. The higher the inter-agency variation relative to total variation, the more agency heterogeneity rather than individual or job characteristics explains staff turnover. Furthermore, if inter-agency variation increases in the first year of a new administration, this would suggest that patronage drives differences in agency turnover, consistent with an “islands of excellence” framework in which some agencies are more subject to political influence than others.

Staff turnover within each agency varies randomly over time. Let *Y*_*ij*_ denote the staff turnover for agency *i* in year *j*. This model can be written as:

$$\begin{array}{c} Y_{ij}=\alpha_{0}+u_{i}+\epsilon_{ij}\;\text{with}\;u_{i}∼N\left(0,\sigma_{u}^{2}\right)\;\text{and}\;\epsilon_{ij}∼N\left(0,\sigma^{2}\right) \end{array}$$ (1)

The *Unconditional Mean Model* can be fitted to the staff turnover data to derive estimates of three model parameters. These include:

- The mean staff turnover across all agencies and years is denoted as ${\hat{\alpha }}_{0}$.
- The variance in within–agency deviations of individual yearly staff turnover, represented by ${\hat{\sigma }}^{2}$.
- The variance in between–agency deviations of agency means from the overall mean across all agencies and years, expressed as ${\hat{\sigma }}_{u}^{2}$.

Additionally, we calculate the intraclass correlation coefficient $\hat{\rho }$ using the formula $\hat{\rho }=\frac{{\hat{\sigma }}_{u}^{2}}{{\hat{\sigma }}_{u}^{2}+{\hat{\sigma }}^{2}}$.

#### The unconditional growth model

This model introduces time as a predictor, and it aims to assess trends in agency turnover over time. We can distinguish between the first year of a new administration and other years, and estimate separate trends.

Let *Y*_*ij*_ be the staff turnover of the *i*^*th*^ agency in the year *j*. Then we can model the linear change in staff turnover over time for agency *i* according to the following model.

$$Y_{ij}=a_{i}+b_{i}{\text{Year}}_{ij}+\epsilon_{ij}\;\text{where}\;\epsilon_{ij}∼N\left(0,\sigma^{2}\right)$$

The parameters in this model denoted as *a*_*i*_, *b*_*i*_, and *σ*^2^, can be estimated through Linear Least Square Regression (LLSR) methods. Here, *a*_*i*_ represents the true intercept for agency *i*, which signifies the expected staff turnover at the start of the observation period (2006). *b*_*i*_ represents the true slope for agency *i*, indicating the expected yearly rate of change in staff turnover for that agency over the electoral years. The *ϵ*_*ij*_ terms denote the deviation of the agency *i*’s actual staff turnover from the expected values under linear growth, specifically the part of the agency *i*’s staff turnover at year *j* that linear changes cannot explain over time. The variability in these deviations from the linear trend is captured by *σ*^2^. In summary, the parameters are:

$$\begin{array}{ccc} a_{i} & : & \text{Intercept}\;\text{for}\;\text{agency}\;i\\ b_{i} & : & \text{Slope}\;\text{for}\;\text{agency}\;i\\ \sigma^{2} & : & \text{Variance}\;\text{in}\;\text{deviations}\;\text{from}\;\text{the}\;\text{linear}\;\text{trend} \end{array}$$

In a multilevel model, we let intercepts *a*_*i*_ and slopes *b*_*i*_ vary by agency and build models for these intercepts and slopes using agency-level variables at level two. An unconditional growth model features no predictors at level two and can be specified either using formulations at both levels:

Level 1:

$$Y_{ij}=a_{i}+b_{i}{\text{Year}}_{ij}+\epsilon_{ij}$$

Level 2:

$$\begin{array}{ccc} a_{i} & = & \alpha_{0}+u_{i}\\ b_{i} & = & \beta_{0}+v_{i} \end{array}$$

or as a composite model:

$$\begin{array}{c} Y_{ij}=\alpha_{0}+\beta_{0}{\text{Year}}_{ij}+u_{i}+v_{i}{\text{Year}}_{ij}+\epsilon_{ij} \end{array}$$ (2)

where *ϵ*_*ij*_ ∼ *N*(0, *σ*^2^) and

$$\left[\begin{array}{c} u_{i}\\ v_{i} \end{array}]∼N([\begin{array}{c} 0\\ 0 \end{array}],[\begin{array}{cc} \sigma_{u}^{2} & \sigma_{uv}\\ \sigma_{uv} & \sigma_{v}^{2} \end{array}]\right)$$

As before, *σ*^2^ quantifies the within-agency variability (the scatter of points around the agency’ staff turnover linear growth trajectories), while now the between-agencies variability is partitioned into variability in initial status $\sigma_{u}^{2}$ and variability in rates of change $\sigma_{v}^{2}$.

#### The conditional growth model

This model introduces level–two predictors and seeks to establish whether important covariates help explain agency variability. Based on previous research [53], we identify two important covariates: the proportion of temporary contracts within the agency (as this contract is more unstable), and the proportion of professionals within the agency (as this rank is large and relatively unstable—managers are also unstable, but are very few). Therefore, the primary aim of this model is to determine whether these covariates significantly explain staff turnover, or if random effects remain significant even after accounting for these compositional factors. An additional aim is to explore whether different types of agencies—those with higher temporary contracts or higher professional staff—have had a different turnover evolution over time.

The annual professional proportion of an agency is defined as the total number of professional public servants in the agency during the year, divided by the agency’s total staffing for that year.

We introduce a categorical variable to classify the “professional” agencies. The median (*Q*_2_) of the professional density distribution across all agencies during the follow-up period, calculated annually, serves as the threshold to classify agencies as either professional or non-professional. From a modeling perspective, we construct a two-level hierarchical model system:

$$\begin{array}{ccc} a_{i} & = & \alpha_{0}+\alpha_{1}{\text{Professional}}_{i}+u_{i}\\ b_{i} & = & \beta_{0}+\beta_{1}{\text{Professional}}_{i}+v_{i} \end{array}$$

Where Professional_*i*_ = 1 if agency *i* is a “professional agency” if the annual average proportion of professionals exceeds the median $Q_{2}^{\left(P\right)}$, calculated from the distribution of the professional density across all agencies for each year, and Professional_*i*_ = 0 if otherwise. In addition, the error terms at level two are assumed to follow a multivariate normal distribution.

Using a binary predictor at level two, such as agency professional status (as an example of variables that characterize the agency), we can define our Level Two Model to differentiate between non–professional and professional agencies.

For non–professional agencies:

$$\begin{array}{ccc} a_{i} & = & \alpha_{0}+u_{i}\\ b_{i} & = & \beta_{0}+v_{i}, \end{array}$$

For professional agencies:

$$\begin{array}{c} a_{i}=\left(\alpha_{0}+\alpha_{1}\right)+u_{i}\\ b_{i}=\left(\beta_{0}+\beta_{1}\right)+v_{i} \end{array}$$

Writing the level two model in this manner helps us interpret the model parameters from our two-level model. We use statistical software (the *lmer*() function from the **lme4** package in R [54]) to obtain parameter estimates using our data, after first converting our level one and level two models into a composite model with fixed effects and random effects separated:

$$\begin{array}{ccc} Y_{ij} & = & a_{i}+b_{i}{\text{Year}}_{ij}+\epsilon_{ij}\\ & = & \left(\alpha_{0}+\alpha_{1}{\text{Professional}}_{i}+u_{i}\right)+\left(\beta_{0}+\beta_{1}{\text{Professional}}_{i}+v_{i}\right){\text{Year}}_{ij}+\epsilon_{ij}\\ & = & [\alpha_{0}+\beta_{0}{\text{Year}}_{ij}+\alpha_{1}{\text{Professional}}_{i}+\beta_{1}{\text{Professional}}_{i}\times {\text{Year}}_{ij}]+\\ & \; & \left[u_{i}+v_{i}{\text{Year}}_{ij}+\epsilon_{ij}\right] \end{array}$$ (3)

We now incorporate the second covariate. The employment of public officials in the Chilean central state is structured around three primary contractual regimes: “Temporary”, “Yearly Contract” (“Yearly” for short), and “Permanent”. Over the 172-month follow-up period, there has been a notable shift in agency composition towards “Yearly” as the preferred contractual arrangement, at the expense of “Permanent” and “Temporary” positions.

**Fig 3:**
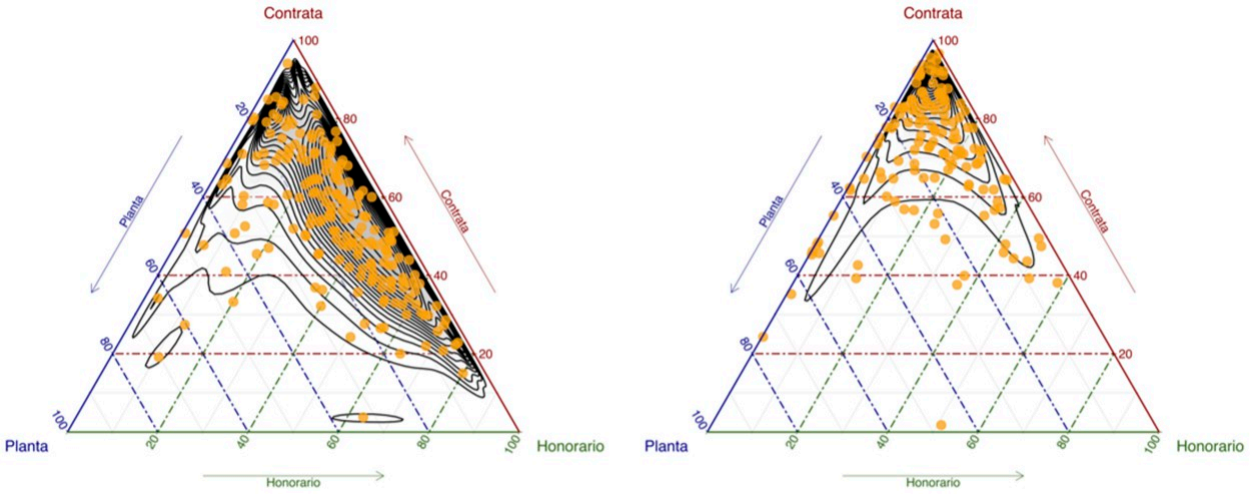
Service composition according to the contracting regimes. The figures are ternary diagrams that illustrate the composition of 269 State agencies according to their contracting regimes. Each point represents an agency, with its position defined by the proportion of officials under “Permanent” (Planta), “Temporary” (Honorarios), and “Yearly” (Contrata) regimes. Panel (a) shows the state of affairs in 2015, while panel (b) depicts 2020. A clear trend toward the “Yearly” regime can be observed. (Source: authors).

Fig 3 depicts the distribution of 269 agencies across these contractual types using ternary diagrams. Panel (a) illustrates this distribution in 2015, where agencies are predominantly situated between “Yearly” and “Temporary”. In panel (b), representing 2020, a consistent trend toward “Yearly” as the favored contractual type is evident. Officials under a “Yearly” arrangement have much higher job stability than those under temporary contracts, yet may exhibit slightly lower stability (for comparable positions) than officials under the “Permanent” type of contract [53].

In our full model, we, therefore, include the proportion of temporary staff in the service as a level–two covariate for determining intercepts and slopes in the model. We define the binary variable *TemporaryStaff* analogously to the Professional binary variable, with the median yearly proportion of Temporary staff across agencies as the cutoff point. Thus, when an agency is above the yearly median proportion, the variable TemporaryStaff = 1, and TemporaryStaff = 0 if otherwise.

Level 1:

$$Y_{ij}=a_{i}+b_{i}{\text{Year}}_{ij}+\epsilon_{ij}$$

Level 2:

$$\begin{array}{ccc} a_{i} & = & \alpha_{0}+\alpha_{1}{\text{Professional}}_{i}+\alpha_{2}{\text{TemporaryStaff}}_{i}+u_{i}\\ b_{i} & = & \beta_{0}+\beta_{1}{\text{Professional}}_{i}+\beta_{2}{\text{TemporaryStaff}}_{i}+v_{i} \end{array}$$

or as a composite model:

$$\begin{array}{ccc} Y_{ij} & = & \alpha_{0}+\alpha_{1}{\text{Professional}}_{i}+\alpha_{2}{\text{TemporaryStaff}}_{i}+\beta_{0}{\text{Year}}_{ij}\\ & + & \beta_{1}{\text{Professional}}_{i}\times {\text{Year}}_{ij}+\beta_{2}{\text{TemporaryStaff}}_{i}\times {\text{Year}}_{ij}\\ & + & u_{i}+v_{i}{\text{Year}}_{ij}+\epsilon_{ij} \end{array}$$ (4)

where the error terms are defined as in the previous models.

Additionally, a model using the proportion of civil servants on temporary contracts and the proportion of professionals as continuous variables, focused on the first years of new administrations, is constructed and analyzed in the supporting information document S1 File. There, we show that the results of such a model are substantively similar. However, for modeling purposes and to facilitate a clearer interpretation of the results, we have opted to present the categorical models in the main text.

### 3.2 Propensity Score Matching (PSM) and mixed-effects models

Propensity Score Matching (PSM) [23, 55] combined with mixed-effects models offers a robust approach for analyzing the impact of political transitions on turnover in the public sector. PSM controls for observable confounders and reduces selection bias by matching services based on key covariates, such as staffing levels, proportion of temporary contracts, and agency-specific characteristics. This matching process creates a balanced dataset, making it particularly useful when a clear, sharp threshold for treatment—such as in natural experiments—is unavailable. By integrating PSM with mixed-effects models, the analysis captures not only the observable differences between matched services but also accounts for unobserved factors like internal management practices, organizational culture, or historical legacies. These latent factors may independently influence turnover, beyond the immediate political transitions. The use of linear mixed-effects models allows for the partitioning of variance into within-service and between-service components, which helps address service-specific heterogeneity—an advantage that other methods, such as Regression Discontinuity Design (RDD) [56], does not inherently provide.

In other words, PSM combined with mixed-effects models provides a flexible and generalizable framework. It allows for the estimation of the average treatment effect (ATE) across a wide range of services while accounting for unobserved heterogeneity through random effects. This approach offers an understanding of how political transitions influence turnover across the entire spectrum of public agencies, making it particularly well-suited for analyzing bureaucratic dynamics across a large number of agencies.

#### Propensity score estimation

We used a logistic regression model to estimate the propensity score for each service. The propensity score represents the conditional probability that a service *i* is exposed to the treatment (i.e., government change), given its observed characteristics:

$$\begin{array}{ccc} \text{log}\;(\frac{P(T_{i}=1)}{1-P(T_{i}=1)}) & = & \beta_{0}+\beta_{1}\cdot {\text{Staffing}}_{i}+\beta_{2}\cdot {\text{Prop\_temporary}}_{i}\\ & & +\beta_{3}\cdot {\text{Prop\_professional}}_{i}+\beta_{4}\cdot {\text{Prop\_contractual}}_{i}\\ & & +\beta_{5}\cdot {\text{Prop\_male}}_{i}+\beta_{6}\cdot {\text{Prop\_managerial}}_{i}\\ & & +\beta_{7}\cdot {\text{Prop\_administrative}}_{i} \end{array}$$ (5)

Where *P*(*T*_*i*_ = 1) is the propensity score for service *i* given its observed characteristics.

Once the propensity scores were estimated, we matched treated and control units using nearest neighbor matching. This approach pairs treated units with control units that have similar propensity scores, ensuring that the two groups are comparable in terms of their baseline characteristics.

After matching, we checked the balance between the treated and control groups by comparing the standardized mean differences and variance ratios for the key covariates. A well-balanced sample indicates that any remaining differences between treated and control groups are minimal.

#### Linear mixed-effects model for matched data: Accounting for the impact of government change on turnover

After estimating the propensity scores and matching the control and treated units, we applied a linear mixed-effects model to the matched data to assess the impact of the treatment (government change) on turnover, while controlling for other relevant covariates. The model incorporated random effects at the service level, which allowed us to account for unobserved heterogeneity across services.

The following mixed effects model was used to analyze the impact of government transitions on employee turnover:

$$\begin{array}{ccc} Y_{ij} & = & \beta_{0}+\beta_{1}\cdot {\text{Treatment}}_{ij}+\beta_{2}\cdot {\text{Staffing}}_{ij}+\beta_{3}\cdot {\text{Prop\_temporary}}_{ij}\\ & & +\beta_{4}\cdot {\text{Prop\_professional}}_{ij}+\beta_{5}\cdot {\text{Prop\_contractual}}_{ij}+\beta_{6}\cdot {\text{Prop\_male}}_{ij}\\ & & +\beta_{7}\cdot {\text{Prop\_managerial}}_{ij}+\beta_{8}\cdot {\text{Prop\_administrative}}_{ij}\\ & & +u_{i}+\varepsilon_{ij} \end{array}$$ (6)

where:

- *Y*_*ij*_: Turnover rate for service *i* at time *j*.
- Treatment_*ij*_: Binary variable indicating whether service *i* is in the treatment group at time *j* (e.g., after a government change)
- *u*_*i*_: Random effect for service *i*, capturing unobserved differences between services, assumed to follow $u_{i}∼N\left(0,\sigma_{\text{Service}}^{2}\right)$.
- *ε*_*ij*_: Residual error for service *i* at time *j*, representing additional unobserved variability, assumed to follow $\varepsilon_{ij}∼N\left(0,\sigma_{\text{residual}}^{2}\right)$.

### 3.3 Mixed Cox survival models for staff longevity

Unlike turnover, which is a property of agencies, longevity is a property of individual bureaucrats. Through an analysis of longevity, we aim to explore the factors that influence the duration of bureaucrats’ careers within state agencies, offering insights into how both individual-level and agency-level characteristics affect employment stability. Longevity analysis is particularly valuable because it allows us to capture long-term dynamics that may not be immediately evident from turnover data alone. While turnover reflects the aggregate effect of staffing changes at the agency level, longevity offers a more granular view of the stability experienced by individual employees. By applying mixed-effects Cox survival models [49], we account not only for the fixed effects of observable covariates such as staff composition and political cycles, but also for random effects that capture unobserved agency-specific factors, such as internal organizational culture or managerial practices, that may influence an employee’s decision to remain within an agency. This approach allows us to quantify the heterogeneity in staff longevity across different agencies while controlling for both observed and latent factors.

When we apply hazard analysis to study public officials’ longevity, we consider the “failure time” as the time that elapsed from the official being part of the agency until she left it. The follow-up time is between January 2006 and April 2020. The event that produces the “failure” is the act of leaving the agency. Some of the officials considered in the follow-up time of this study do not leave the agency, so their survival time is not known with certainty, and the observations are right–censored. For the analysis, we take into account: 1) individuals do not enter the study (date of entry into the agency) at the same time, and 2) not all officials leave the agency (“failure” event) during the follow-up time. The great advantage of survival models is that these cases are considered appropriately.

Staffing data exhibits a hierarchical structure, with individuals nested within agencies. In hazard analysis, it is imperative to acknowledge the correlations among individuals within these hierarchical structures. Survival outcomes within one agency may systematically differ from those in another agency under similar conditions, a phenomenon often attributed to unobserved latent factors inducing “homogeneity” within agencies.

To address this, we employ mixed models, encompassing both fixed and random effects. Specifically, in this study, we use a single random intercept. As proposed by Crowther et al. [57], such models are referred to as “frailty models”, distinguishing them from models with multiple random effects. To capture the influence of agency-specific factors on officials’ survival, we adopt “shared frailty” models. These models incorporate random effects into the Cox model, resulting in varying risk levels among agencies– hence the term “frailty” denoting the added risk.

The random effect, akin to a random intercept for each agency, adjusts the linear predictor in the hazard function’s exponent. Consequently, the shared frailty term introduces a multiplicative effect on the base risk rate, leading to inter-agency variability in survival outcomes. Agencies with higher fragility values are expected to experience failures earlier than those with lower fragility values, serving as a proxy for unobserved agency effects.

Cox regression models with mixed effects are distinguished by the distribution of shared frailty terms. Various distributions have been proposed, with our study opting for the normal distribution (Gaussian) to characterize the shared frailty terms.

Formally, to account for random effects, the Cox model is modified this way:

$$h\left(t|X,Z\right)=h_{0}\left(t\right)e^{X\beta +Zb}$$

b∼G(0,(θ))

Where *h*_0_ is the unspecified base hazard function, *X* and *Z* are the design matrices for the fixed and random effects respectively, *β* is the vector of coefficients for the fixed effects, and *b* is a vector of coefficients for the random effects. The random effects distribution is modeled with a Gaussian distribution *G* with zero mean and variation matrix *Σ*, which depends on the parameter *θ*. In the model, *Z*_*ij*_ = 1 if the official *i* is part of the agency’s staff *j*. If *N* is the number of agencies, then there are *N* coefficients *b* for the random effects, and it is true that ∑_*p*_ *b*_*p*_ = 0. Usefully, and unlike the case for only fixed effects, it is not required to set a coefficient to zero for one of the agencies that would serve as a reference. The additional risk *f*_*k*_ = exp(*b*_*k*_) for the agency *k* can be considered as a base tendency for the agency to fail. This value can be associated with the agency’s stability and, therefore, with the agency’s capacity and autonomy, as previously discussed.

We will consider two types of models, using individuals as the unit of observation.

i. The model with only agencies as covariates gives us the shared frailty value of each agency. The motivation for this model is that it allows us to obtain a specific frailty value for each agency, enabling us to quantify its longevity risk and observe the extent of heterogeneity between agencies.
ii. We then introduce job-related and individual-related covariates to see whether these help to significantly account for longevity. Job-related characteristics are contract type, rank, and agency size. Individual–related characteristics are sex, age, and age squared. With this model, we can determine whether these characteristics affect longevity risk. Additionally, we can again obtain agency-specific frailties and assess whether they have been significantly influenced by the introduction of these covariates.

### 3.4 Quantifying workforce heterogeneity and stability in public services

The final step in our analysis is to derive indicators for each agency on the two dimensions on employment stability that we have identified: a more short-term view based on the agency’s susceptibility to election-related employment shocks, and the agency’s longer-term latent “risk” or frailty. Both indicators are derived from our mixed-effects models. We formalize this approach by defining the following two key metrics:

*Service Hazard Rate* (SHR_*k*_), derived from the Service frailty:

$${\text{SHR}}_{k}=\text{exp}\left(b_{k}\right)$$

Where *b*_*k*_ represents frailty of the service *k*,

And, the *Relative Turnover Difference* (RTD_*k*_) of the service *k*, derived from the random intercepts for the regular and post-electoral turnover models, and which compares turnover in post-electoral years to regular years:

$${\text{RTD}}_{k}=\frac{{\left(\text{Post-electoral}\;\text{Turnover}\right)}_{k}-{\left(\text{Regular}\;\text{Turnover}\right)}_{k}}{{\left(\text{Regular}\;\text{Turnover}\right)}_{k}}$$

We can then organize the observed agency heterogeneity along these two dimensions through cluster analysis.

## 4 Results

### 4.1 Results for staff turnover

For the staff turnover analysis, we use a sample of 77 State agencies with complete data throughout the monitoring period.

**Fig 4:**
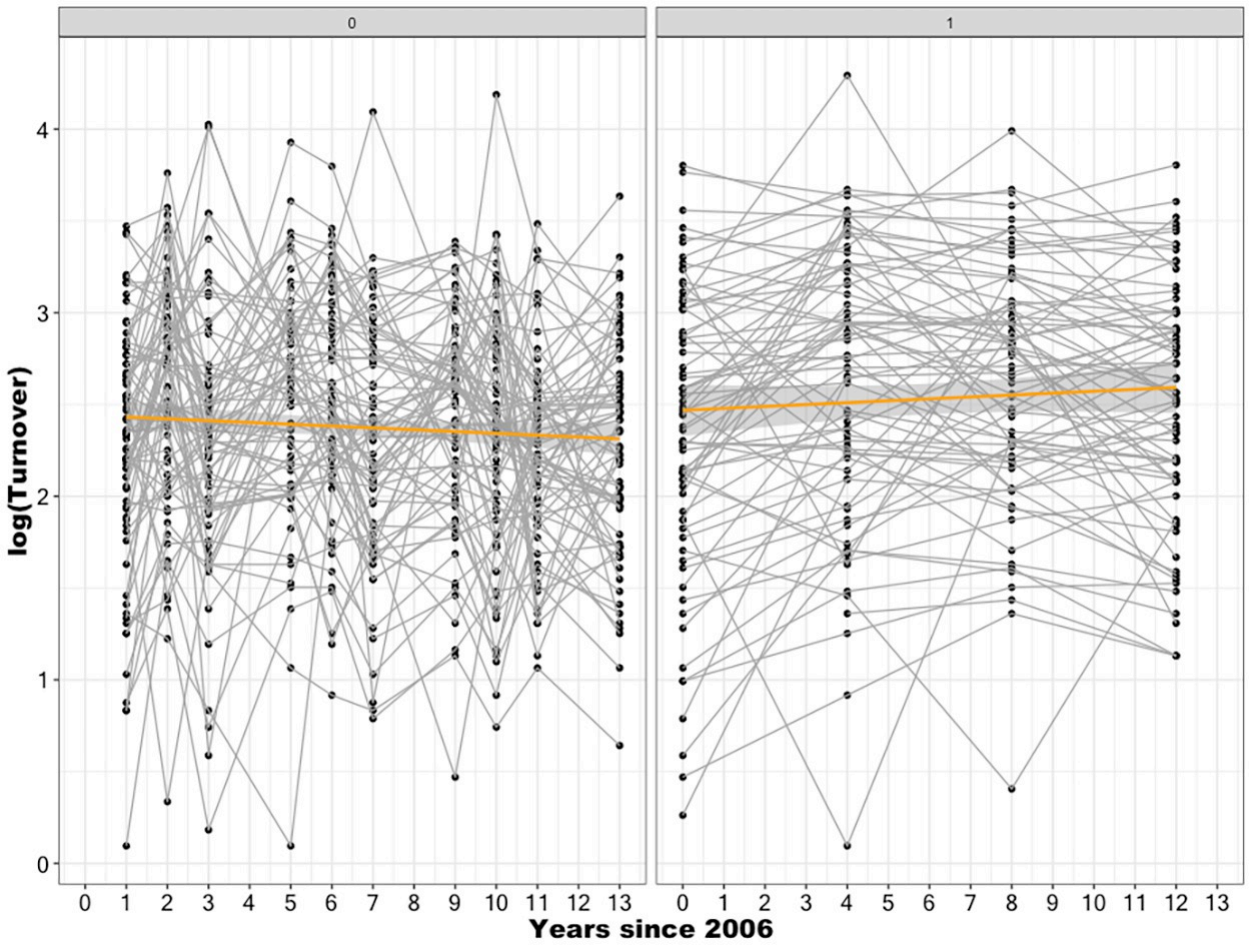
An overview of the staff turnover trends. “Spaghetti plots” illustrating time trends for each agency by year type—regular years (left panel) and the first year of different government administrations (right panel) —for a sample of 79 agencies, with overall linear fits depicted by the bold orange line. (Source: authors).

As an initial overview of turnover dynamics in Chilean central state agencies, Fig 4 plots the average turnover for each service over the years this study covers and draws the trend line. The left panel considers regular years, while the right panel shows staff turnover in the first years of respective government changes.

In regular years, staff turnover remains practically constant, with an average value of around 12% throughout the monitoring period. Staff turnover behaves like a random variable influenced by many random events, making its value consistent. As Talagrand states in [58], this phenomenon can be described as follows: a random variable that depends (in a “soft” way) on the influence of many independent variables (but not too much on any single one) is essentially constant. When considering only the first years of government changes, central government agencies appear to have a very slowly increasing turnover over time (see the right panel of Fig 4). However, as we will see later, the estimate of this increase using the *Unconditional Growth Model* shows that this apparent increase is not statistically significant. Importantly, Fig 4 also shows significant turnover variability between agencies every year.

To analyze these results systematically, we present the results for each model, in the order they were presented in the previous section.

#### Unconditional mean model results

Recall that the main goal of the unconditional means model 1 is to analyze how much variation in staff turnover occurs within agencies and how much takes place between agencies. We estimate separately the model for post-electoral and for regular years; for simplicity, we focus here on post-electoral year results. Applied to the staff turnover data, it yields the following estimates for the three model parameters:

- The mean turnover across all agencies and years: ${\hat{\alpha }}_{0}=15.94$.
- The variance in within-agency deviations between individual yearly turnover and the agency mean: ${\hat{\sigma }}^{2}=34.38$.
- The variance in between–agency deviations between agency means and the overall mean: ${\hat{\sigma }}_{u}^{2}=64.13$.

The intraclass correlation coefficient, $\hat{\rho }$, is calculated as $\frac{{\hat{\sigma }}_{u}^{2}}{{\hat{\sigma }}_{u}^{2}+{\hat{\sigma }}^{2}}$, resulting in $\hat{\rho }=\frac{64.13}{64.13+34.38}\approx 0.65$. This indicates that 65% of the total variation in staff turnover during the first year of new administrations can be attributed to differences among agencies, rather than changes over time within the agencies. This reaffirms the heterogeneity of state services in their relationship with the political cycle, suggesting varying degrees of autonomy. For comparison, the unconditional mean model for regular years shows that inter-agency variation accounts for 39% of staff turnover, with 61% being accounted for intra-agency variation (the values are ${\hat{\alpha }}_{0}=12.53$ and $\hat{\rho }\approx 0.39$).

These results indicate that both individual and agency-specific factors influence turnover. However, the finding that nearly 40% of the variation in turnover during “non-political” years is due to differences between agencies highlights substantial heterogeneity in employment stability, which affects agency capacity through the channels discussed above. Furthermore, in the first year of a new government, differences between agencies account for 65% of the variation, strongly suggesting that politicians differentially target agencies for employment purposes. While in some cases this may be for reasons of policy control (for instance, in undersecretaries) and in others simply to reward co-partisans (possibly, in agencies with non-critical missions such as the *National Youth Institute*), the fact remains that the effect is not proportional to all agencies. This implies that some agencies are significantly more autonomous than others. For instance, the *Tax Agency* and *the Budget Agency*—two crucial agencies for the well-functioning of the state as a whole—exhibit remarkable independence from political cycles.

#### Unconditional growth model results

The main goal of this model is to estimate time trends in turnover. Using the composite model 2, we applied the unconditional growth model to analyze staff turnover across state agencies, focusing specifically on post-electoral years. Table 1 presents the estimates for the six key model parameters.

**Table 1:** Unconditional growth model estimates for post-electoral staff turnover.

Parameter	Estimate	Description
${\hat{\alpha }}_{0}$	15.28	Mean turnover in 2006
${\hat{\beta }}_{0}$	0.18	Mean yearly change in turnover
${\hat{\sigma }}^{2}$	27.97	Within-agency variance
${\hat{\sigma }}_{u}^{2}$	75.46	Between-agency variance (2006)
${\hat{\sigma }}_{v}^{2}$	0.23	Between-agency variance in rate changes
${\hat{\rho }}_{uv}$	-0.38	Correlation between 2006 turnover and rate change

The mean staff turnover in 2006 was 15.28% (95% CI: [12.92, 17.65]), with an annual increase of 0.18%, although this increase was not statistically significant (${\hat{\beta }}_{0}=0.184$, 95% CI: [−0.08, 0.29], *p* = 0.246; Std. ${\hat{\beta }}_{0}=0.05$).

Turnover rate variation among agencies was 23%. A moderate negative correlation of -0.38 indicates that agencies with higher turnover in 2006 saw a moderate reduction in turnover rate changes, suggesting increased stability over time.

The within-agency variance, ${\hat{\sigma }}^{2}$, decreased by 19% compared to the *Unconditional Means Model*, showing that 19% of within-agency turnover variability can be attributed to linear trends over the years:

$$\text{Pseudo}\;R^{2}=\frac{{\hat{\sigma }}_{\text{uncond}\;\text{means}}^{2}-{\hat{\sigma }}_{\text{uncond}\;\text{growth}}^{2}}{{\hat{\sigma }}_{\text{uncond}\;\text{means}}^{2}}=\frac{34.38-27.97}{34.38}=0.19$$

**Fig 5:**
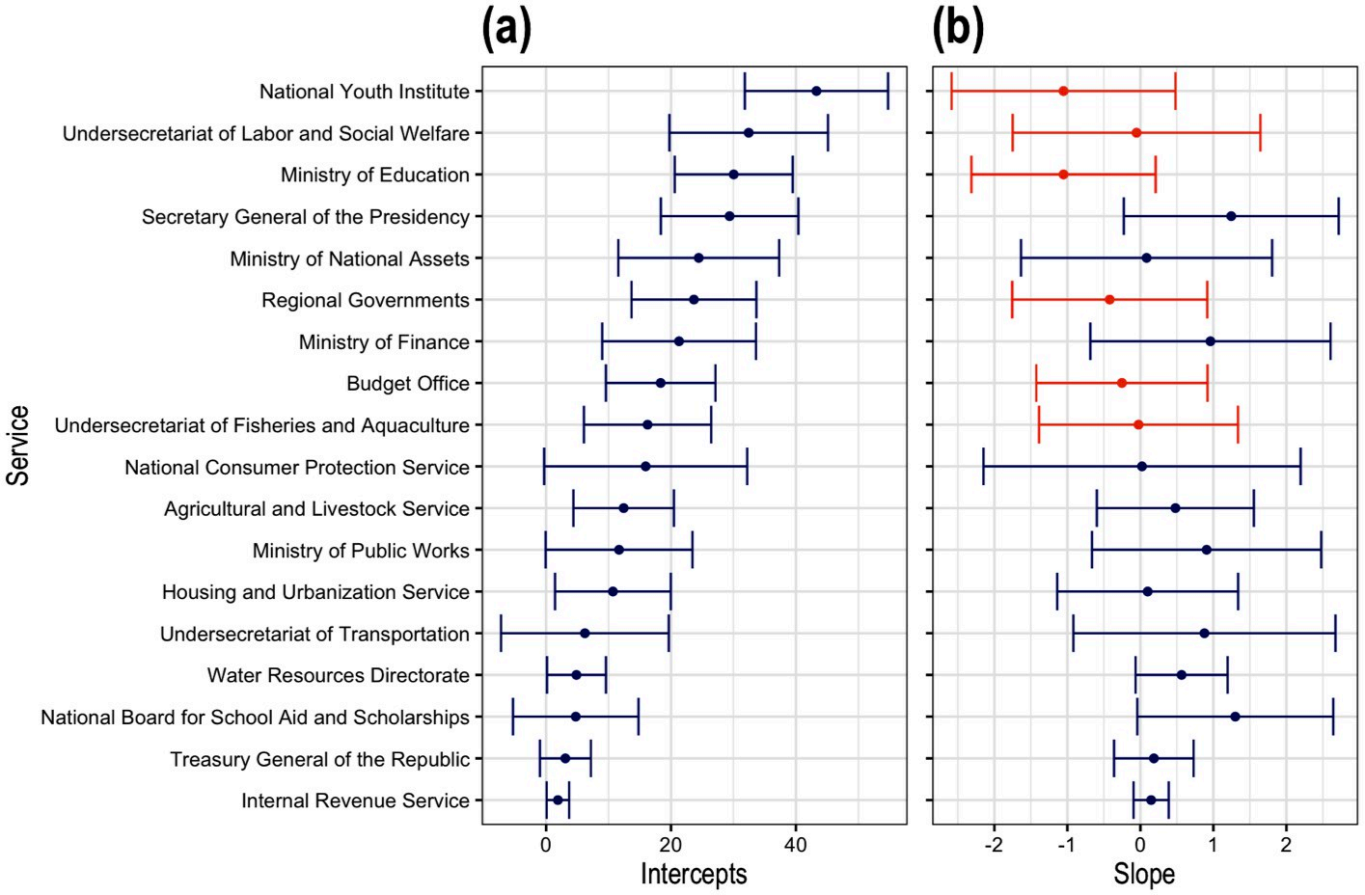
The intercepts and slopes for the unconditional growth model. The figure presents the intercept values *u*_*i*_ (panel a) and slope values *v*_*i*_ (panel b) with error bars for each agency *i*, derived from fitting the *Unconditional Growth Model*. For clarity, only twenty services are displayed. (Source: authors).

Although the turnover trends were not strictly linear, the linear model provides a simple and reasonable approximation of individual agency trends. The *Unconditional Growth Model* confirms that base turnover in 2006 varied across agencies, but the rate of change (slopes) was not significantly different from zero, indicating stable turnover patterns over time (see Fig 5).

A model with quadratic terms for time reveals small but statistically significant quadratic effects on turnover (details in supporting information S1 File).

In summary, the results from the unconditional growth model suggest that, despite three consecutive changes of government, the overall turnover levels within the State remained remarkably stable over time. This stability persists even in electoral years, as the upward trend in turnover was statistically insignificant, indicating a political equilibrium where each new administration replaces a consistent but limited number of positions without escalation. The observed trend—where agencies with initially high turnover rates reduced turnover, while those with lower rates experienced slight increases—suggests a gradual convergence in turnover patterns across agencies, indicating a reduction in agency heterogeneity over time.

#### The conditional growth model results

In the previous models, we observed that a significant proportion of the variance in turnover during the first year of new administrations is attributable to persistent differences between services. These differences remain consistent over time, suggesting that state turnover rates are stable, with no significant fluctuations during the observation period.

In the subsequent model, still focusing on post-electoral years, we introduce controls for two key service characteristics to assess their role in explaining these differences. Specifically, we account for the *proportion of professionals* and the *contractual regime* to capture the influence of these variables on turnover dynamics.

The results for the “intermediate model” 3, which includes only the binary variable *Professional*, can be found in the supporting information S1 File. Here, we present the results for the model 4 that includes both binary variables, *Professional* and *TemporaryStaff*, which are binarized according to the procedure outlined above.

Table 2 presents the results from fitting the Conditional Growth Model 4, incorporating both fixed and random effects, with the covariates *Year*, *Professional*, and *TemporaryStaff*.

**Table 2:** Results of the conditional growth model with professional and temporary staff status.

**Fixed Effects**	**Estimate**	**95% CI**	**p–value**
${\hat{\alpha }}_{0}$ : Baseline turnover (2006)	9.15	[6.56, 11.75]	<.001
${\hat{\alpha }}_{1}$ : Professional agencies (low temp. staff)	4.69	[2.00, 7.37]	<.001
${\hat{\alpha }}_{2}$ : High Temp. agencies (low professional staff)	8.44	[5.64, 11.23]	<.001
${\hat{\beta }}_{0}$ : Annual turnover increase (non-professional, low temp. staff)	0.44	[0.13, 0.75]	0.006
${\hat{\beta }}_{1}$ : Year × Professional	-0.46	[-0.81, -0.12]	0.009
${\hat{\beta }}_{2}$ : Year × Temporary Staff	-0.03	[-0.38, 0.32]	0.859
**Random Effects**	**Variance**	**Std. Deviation**	
*σ*^2^: Within-agency variance	21.59	4.64	
$\sigma_{\text{Service}}^{2}$ : Inter-agency variance	43.26	6.58	
$\sigma_{\text{Service}-\text{Year}}^{2}$ : Service-Year interaction	0.10	0.32	
*ρ*_Service- Year_: Correlation (service × year)	-0.26		
$\hat{\rho }$ : Intraclass Correlation Coefficient	0.66		
**Model Statistics**			
Conditional *R*^2^	0.73		
Marginal *R*^2^	0.20		

The fixed effects show that baseline turnover in 2006 is estimated at 9.15%. We can see that both added dummy variables are statistically significant and add substantial turnover to the baseline: Professional agencies exhibit turnover 4.69 percentage points higher, while agencies with high proportion of temporary staff have an 8.44% higher turnover—a substantial amount. Consistent with the previous results, time trends are small in magnitude. Non-professional agencies with low temporary staff experiencing a slight increase in turnover, and professional agencies experiencing a small decrease. The turnover rate for agencies with higher temporary staff remains stable over time.

The random effects indicate substantial inter-agency variance (43.26%) and a small variance component for year-related trends (0.10%), with a 66% intraclass correlation coefficient, again highlighting the importance of agency-specific factors in explaining turnover variability.

The model demonstrates strong explanatory power, with a conditional *R*^2^ = 0.73, indicating that 73% of the variance in turnover is accounted for when considering both fixed and random effects. However, the marginal *R*^2^ of 0.2—reflecting the explanatory contribution of the covariates alone—suggests that while these factors are statistically significant, they explain only a modest portion of the overall variance, highlighting the critical role of unobserved, service-specific factors captured by the random effects.

In summary, the conditional growth model reveals that the presence of professional and temporary staff plays a significant role in shaping turnover rates within Chilean state agencies during post-electoral years.

The random intercepts remain highly significant, indicating that underlying differences in agency politicization are substantial and are not fully explained by the fixed effects of the covariates included in the model. Additionally, the intraclass correlation coefficient (ICC), which attributes 66% of the total variance in post-electoral years to differences between agencies, remains virtually unchanged from the unconditional mean model. This suggests that the introduction of time trends and covariates did not significantly alter the agency-specific variance. These findings underscore the strong influence of agency-specific characteristics on turnover.

#### Causal inference with propensity score matching and mixed-effects models

**Fig 6:**
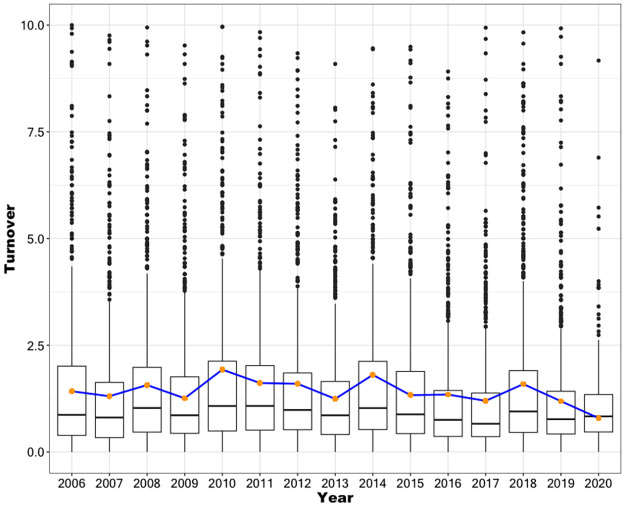
Average annual staff turnovel. The figure shows the annual turnover distributions for 77 services of the Central Chilean State. The blue line and orange dots indicate the evolution of the average annual turnover values. (Source: authors).

For this analysis, we used turnover records from 77 Central Government services covering the period from January 2006 to April 2020, during which four changes of administration occurred (in March 2006, 2010, 2014, and 2018), though the first was within the same political coalition. Fig 6 presents the turnover distributions across this timeframe. Notably, an increase in the mean turnover is observed in the years following each government transition between rival coalitions (2010, 2014, and 2018), corresponding to the installation of new administrations.

The impact of these changes on turnover was analyzed using a two-step methodology. First, we estimated the propensity score through a logistic regression model to control for potential confounding factors. Then, we applied a mixed-effects model to assess the treatment effect, incorporating random effects at the service level to account for unobserved heterogeneity between services.

**Table 3:** Results of the mixed linear model on matched data: Turnover analysis with fixed and random effects.

**Fixed Effects**	**Estimate**	**Std. Error**	**t value**	**Pr(> |*t*|)**
(Intercept)	1.321	0.089	14.829	0.00
treatment	0.507	0.109	4.659	0.00
staffing_scaled	0.044	0.082	0.535	0.59468
Prop_temporary_scaled	1.019	0.122	8.382	0.00
Prop_professional_scaled	0.054	0.090	0.603	0.54887
Prop_contractual_scaled	0.121	0.112	1.073	0.28754
Prop_male_scaled	0.011	0.078	0.139	0.88971
Prop_managerial_scaled	0.200	0.077	2.605	0.00993
Prop_administrative_scaled	0.089	0.083	1.072	0.28905
**Random Effects**	**Variance**	**Std.Dev.**		
Service (Intercept)	0.1313	0.3624		
Residual	5.2968	2.3015		

Here, we present the results based on records from January 2015 to November 2017, representing the control group. This period is considered regular, without any change in administration. The treated group includes the same services from December 2017 to December 2018, coinciding with the installation of the new administration. We include in this period the three months prior to the March 2018 transition (following the December 2017 presidential run-off) to capture potential “anticipatory” turnover. The variable “treatment” is a binary indicator reflecting whether the observation occurred during the treatment period. To ensure comparability across variables, all covariates were scaled before fitting the model.

#### Propensity score matching results

To ensure comparability between the treated and control groups, we applied propensity score matching using the nearest neighbor method. Before Matching, there was a notable imbalance between groups. For instance, the standardized mean difference for Prop_temporary was -0.3555, indicating a significantly lower proportion of temporary staff in the treated group. The variance ratios and eCDF metrics also showed considerable discrepancies.

After Matching, the standardized mean differences were reduced to near zero (e.g., Prop_temporary -0.0084), and variance ratios improved, indicating strong balance between the groups. The eCDF Mean dropped from 0.0943 to 0.0008, further confirming distributional alignment. These results confirm that the propensity score matching effectively balanced key covariates, allowing for a more reliable comparison between the groups.

#### Results of the mixed linear model applied to the matched data

The results of the mixed linear model applied to the matched data, obtained through propensity score matching (see Table 3), provide valuable insights into the factors driving staff turnover in state agencies.

The treatment variable, which represents the effect of the change in government, has a statistically significant positive effect on turnover (*β* = 0.507, *p* = 3.41 × 10^−6^). This suggests that government transitions are associated with an increase of about half a standard deviation in staff turnover, confirming the disruption often caused by political shifts.

Among the covariates, the proportion of temporary staff (*Prop_temporary_scaled*) has the strongest and most significant effect on turnover (*β* = 1.019, *p* = 3.10 × 10^−12^). Other covariates, such as *staffing*, *proportion of professionals*, *proportion of contractual officials*, and *proportion of male employees*, were not statistically significant, indicating that their influence on turnover may be limited in this context. However, the *proportion of managerial staff* (*Prop_managerial_scaled*) has a statistically significant positive effect on turnover (*β* = 0.200, *p* = 0.00993). This suggests that services with a higher percentage of managerial staff experience slightly higher turnover rates during the period analyzed, most likely due to the political exposure of managerial roles.

In summary, the model highlights the importance of both organizational characteristics and service-specific factors in driving turnover, with government transitions and the proportion of temporary staff emerging as the most influential variables. Importantly, the model suggests that the spike in bureaucratic turnover we observe in post-electoral years may indeed be causal.

Finally, in order to better understand the mechanisms driving the relationship between government transitions and staff turnover, we employed mediation analysis [59], using the proportion of temporary contracts as a mediating variable. Our analysis suggests that approximately 21.4% of the total effect of government transitions on turnover is explained through the mediating influence of temporary contract proportions. This suggests that temporary employees are more exposed to turnover risks during political transitions, which in turn amplifies overall turnover. The results underscore the importance of workforce composition, particularly the reliance on temporary contracts, as a key factor in how political shifts affect public sector stability. A full report of these results, as well as a further sensitivity analysis, can be found in supplementary information S1 File.

### 4.2 Results for longevity of state personnel

#### Fixed–effect Cox model results: Service as a covariate

While turnover captures the overall staffing changes at the agency level, longevity provides a detailed perspective on the long-term stability of individual employees within the organization. In this analysis employing a fixed-effect model, we examine a subset of the dataset comprising 77 central State agencies with complete records spanning from January 2006 to April 2020. The longevity of officials within these agencies is computed based on entry and departure dates extracted from this data. Survival status is determined by the departure date, with a status of “1” assigned if the departure date precedes the end date of the follow-up period, and “0” otherwise (implying right-censored data).

**Fig 7:**
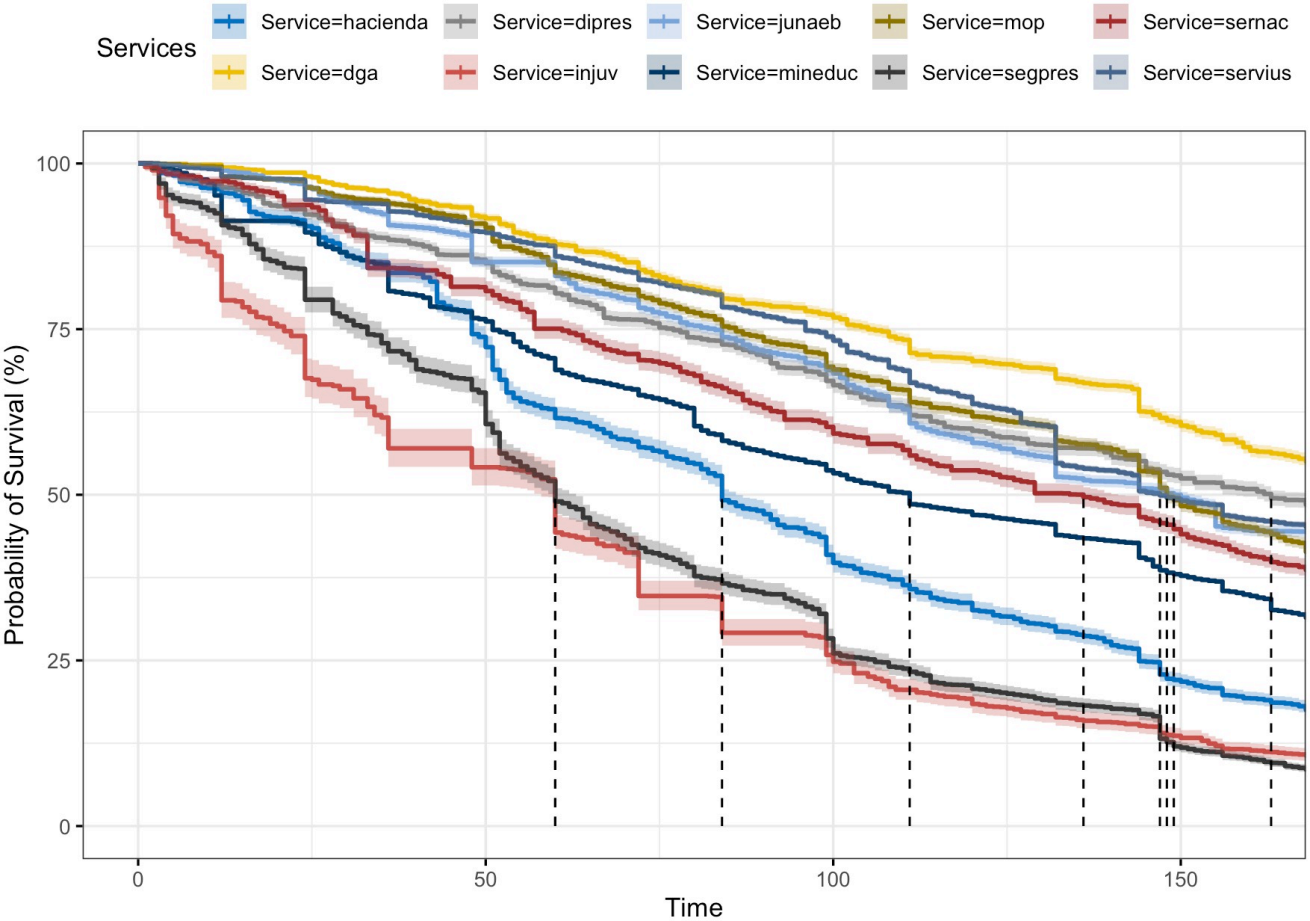
Kaplan—Meier curves stratified by state agencies. The figure displays the survival curves of ten randomly selected agencies from the entire set of state agencies. There are discernible differences in the longevity of these agencies. This pattern is a general characteristic across agencies, not limited to this sample. The dashed lines represent the medians of the survival probability distributions for each agency. The agencies included in the figure, along with their corresponding median longevity (in months), are as follows: DGA: Water Resources Directorate (171), Hacienda: Ministry of Finance (84), JUNAEB: National Board for School Aid and Scholarships (149), MINEDUC: Ministry of Education (111), SERNAC: National Consumer Protection Service (136), SEGPRES: Secretary General of the Presidency (163), INJUV: National Youth Institute (60), MOP: Ministry of Public Works (148), DIPRES: Budget Office (163), and SERVIUS: Regional Housing and Urbanization Services (147).

Fig 7 displays the Kaplan—Meier curves stratified by agency, with only ten agencies randomly selected for better visualization. The aim is to illustrate the variation in civil servants’ longevity across different state agencies. The figure highlights significant discrepancies, which are representative of broader trends, not limited to the depicted sample. The vertical dashed lines denote the medians for the survival probability distributions of each agency, indicating the time within which the survival probability drops to 50%. This metric signifies that half of agency staff have longevity equal to or less than this value.

This analysis reaffirms the idea of important differences in survival probabilities among the various state agencies and suggests that simply belonging to a particular agency may impose certain “constraints” on the longevity of its officials. For the reasons discussed above, agencies with shorter longevity may likely exhibit diminished capacity compared to agencies with higher survival probabilities for their personnel.

Table 4 displays the risk rates obtained through Cox proportional regression for a subset of ten services, using a single predictor variable—the agency, with the *Ministry of Finance* (Hacienda) as the reference. Risk rates below one suggest a decreased risk compared to the *Ministry of Finance*, while rates exceeding one indicate a higher risk (implying officials have shorter longevity). For instance, the *National Youth Institute* (INJUV) and *Secretary General of the Presidency agencies* (SEGPRES) stand out, demonstrating higher risk rates.

**Table 4:** Hazard rates for a sample of state agencies.

Agency ID	HR* (95% CI)	p–value
Ministry of Finance	–	–
Water Resources Directorate	0.40 (0.39 to 0.42)	<0.001
Budget Office	0.42 (0.40 to 0.44)	<0.001
National Youth Institute	1.22 (1.16 to 1.28)	<0.001
National Board for School Aid and Scholarships	0.47 (0.45 to 0.49)	<0.001
Ministry of Education	0.66 (0.64 to 0.68)	<0.001
Ministry of Public Works	0.51 (0.49 to 0.53)	<0.001
Secretary General of the Presidency	1.37 (1.31 to 1.42)	<0.001
National Consumer Protection Service	0.54 (0.52 to 0.57)	<0.001
Regional Housing and Urbanization Services	0.49 (0.47 to 0.51)	<0.001

Hazard rates were estimated using a Cox regression model, where each state agency served as the sole predictor variable, with the Ministry of Finance as the reference category. HR* Instantaneous Hazard Ratio

#### Mixed Cox model results: Considering agency random effects

**Fig 8:**
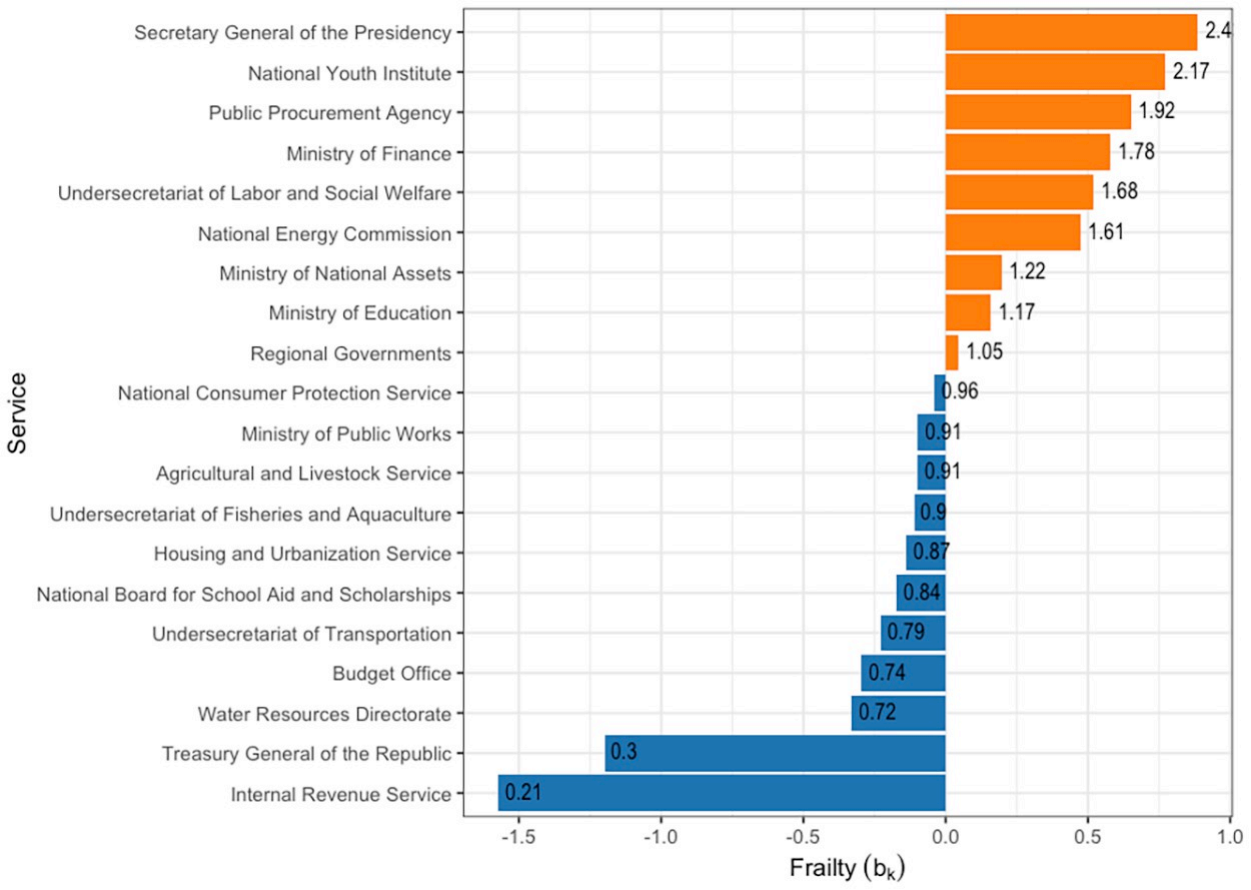
Frailties computed for the mixed Cox model of staff longevity using a sample of twenty agencies. The figure illustrates the frailty values derived from the mixed Cox model, representing the longevity of state officials in a randomly selected sample of twenty state agencies. Each bar label corresponds to the exponent of the random intercept *f*_*k*_ = exp(*b*_*k*_). (Source: authors.).

Public servants work within state agencies, and their longevity is not an independent variable; rather, it is correlated with their colleagues’, influencing survival estimates. In other words, the agency itself plays a role in determining the survival of its officials. The mixed Cox model provides a method to estimate this effect by introducing a random intercept or “shared frailty” term that adjusts the baseline risk according to the agency. Fig 8 visually depicts the differences in shared frailties among state agencies for the longevity of their officials, using a mixed Cox model where the agency serves as the covariate.

The shared frailty values reveal significant disparities among agencies, underscoring the notable heterogeneity within the state structure. For example, certain agencies like the *Internal Revenue Service* (rate = 0.21), *Treasury General of the Republic* (rate = 0.3), and the *Water Resources Directorate* (rate = 0.72) exhibit stability and lower risk compared to higher-risk agencies such as the *Secretary General of the Presidency* (rate = 2.4), *National Youth Institute* (rate = 2.17), and *Public Procurement Agency* (rate = 1.92), among others. The key difference with the results presented in Table 4 is that the risk values (frailties) are not referenced to any specific agency but are instead derived from a broader risk distribution.

An advantage of this model is that we can interpret this estimate akin to other coefficients in the Cox regression. Specifically, by exponentiating the standard deviation (*σ* ≈ 0.516) to yield exp(*σ*) = exp(0.516) ≈ 1.676, we can infer that a state agency with a frailty value one standard deviation above the mean has an approximately 67.6% higher risk compared to the average.

The differences in agency risk are thus substantively quite large. For example, *ChileCompra* (the public procurement agency), with a frailty value of 0.667, has a relative risk of exp(0.667) ≈ 1.95. This indicates that ChileCompra has approximately a 95% higher risk that its employees will leave the service compared to the average. Conversely, the Internal Revenue Service, with a frailty value of −1.62, has a relative risk of exp(−1.62)≈0.198. Therefore, the *Internal Revenue Service* has approximately an 80.2% lower risk of employee departure compared to the average.

#### Results of mixed Cox models: Accounting for covariates

In this section, we analyze the risk of exiting an agency using a Cox mixed model, incorporating several covariates. This model examines the influence of various characteristics of public servants and of the job they are in. Specifically, we control for *sex*, *job rank*, *contractual regime*, *age*, and the *logarithm of service size*. Additionally, the *service* (agency) is treated as a random effect to account for differences between agencies. In this context, the frailty value represents the variability in the risk of turnover that cannot be explained by the covariates.

The model is applied to a representative sample of 77 central government agencies in Chile, covering a 172—month follow-up period.

The results of the mixed-effects Cox model, as shown in Table 5, provide a detailed analysis of the risk factors associated with civil servant exits in Chilean state agencies. Regarding *sex*, the model indicates that female civil servants have a hazard ratio of 0.903, meaning that women have a 9.7% lower risk of leaving their jobs compared to men, the reference group. This result is statistically significant, suggesting a gender disparity in exit risk across agencies.

**Table 5:** Fixed and random effects results for the mixed Cox model.

**Covariable**	***β***	**exp(*β*)**	***p*–value**
Sex = Female	-0.1021	0.9029	< 2 × 10^−16^
Rank = Administrative	-0.1167	0.8899	< 2 × 10^−16^
Rank = Auxiliary	0.0776	1.0810	< 2 × 10^−16^
Rank = Government Authority	1.8140	6.1360	< 2 × 10^−16^
Rank = Inspector	-0.3355	0.7150	< 2 × 10^−16^
Rank = Managerial	0.6746	1.9630	< 2 × 10^−16^
Rank = Technician	-0.2655	0.7668	< 2 × 10^−16^
Rank = Unclassified	0.9380	2.5550	< 2 × 10^−16^
Contract Regime = Permanent	0.3174	1.3730	< 2 × 10^−16^
Contract Regime = Temporary	0.6532	1.9220	< 2 × 10^−16^
Age	-0.0939	0.9104	< 2 × 10^−16^
Age^2^	0.0011	1.0010	< 2 × 10^−16^
Log Service Size	-0.4530	0.6357	< 2 × 10^−16^
**Random Effects**	**Variance**	**Std. Deviation**	
Service (Intercept)	0.2149	0.4636	

The table presents the fixed effects coefficients and random effect variance for the Mixed Cox Model.

For *job rank*, the baseline category is professional employees. Compared to that rank, administrative staff have an 11.0% lower risk of exit (HR = 0.8899), while inspectors and technicians also face reduced risks (HR = 0.715 and HR = 0.767, respectively). Conversely, auxiliary workers are 8.1% more likely to exit (HR = 1.081), and unclassified staff show a 155.5% higher risk (HR = 2.555). More politically exposed ranks have the highest exit risk. Managerial staff are nearly twice as likely to leave (HR = 1.963) than professionals, while government authorities (such as ministers and undersecretaries) are over five times as likely, at 513.6% (HR = 6.136).

In terms of *contractual regime*, permanent civil servants have a 37.3% higher risk of leaving than those on yearly contracts (HR = 1.373), despite the common perception of permanency providing stability. This is most likely explained by the fact that high-risk positions such as government authorities and managerial roles are legally required to hold permanent contracts. Temporary staff face an even greater risk of exit, with a hazard ratio of 1.922, indicating a 92.2% increased risk compared to yearly contract workers.

For *age*, each additional year reduces the hazard rate by approximately 8.96% (HR = 0.9104), suggesting that older employees are less likely to exit. However, the small positive coefficient for *age squared* (HR = 1.0010) indicates a slight increase in risk as individuals age beyond a certain point, with the risk reduction beginning to reverse around age 43, likely due to retirement considerations.

The *log of service size* is associated with greater stability, as larger agencies have lower turnover. Specifically, a one-unit increase in the log of service size reduces the hazard by 36.4% (HR = 0.6357), suggesting that larger services offer more stable environments for civil servants.

When the model excludes covariates, the variance for service frailties is 0.266, and after including all covariates, the variance reduces only slightly to 0.215. This 19.3% reduction indicates that while the covariates explain some of the differences between services, a significant proportion of the variance remains unexplained, reflecting agency-specific factors not captured by the individual-level characteristics in the model.

In conclusion, the mixed-effects Cox model highlights significant variability in turnover risk across different job ranks, contractual regimes, and individual characteristics within Chilean state agencies. While the inclusion of covariates provides important insights into these risks, the model underscores the importance of agency-specific factors in shaping turnover, with substantial heterogeneity persisting across services even after accounting for key individual and job-level variables. This suggests that unobserved factors intrinsic to each agency, such as patronage dynamics or management practices, continue to play a critical role in shaping staff stability.

### 4.3 Quantifying agency stability: Clustering based on Service Hazard Rate and relative turnover difference

In section 3.4, we introduce two key quantitative indices to operationalize agency stability: Service Hazard Rate (SHR) and Relative Turnover Difference (RTD). SHR, derived from the frailty value in mixed-effects Cox models, quantifies long-term service stability by capturing latent, service-specific factors influencing staff longevity. Meanwhile, RTD measures short-term disruptions in turnover, particularly those induced by electoral cycles and political transitions. The combination of these indices offers a parsimonious way to assess both structural stability and politically-driven instability within state agencies. A table containing the metrics for Service Frailties, Service Hazard Rate (SHR), and Relative Turnover Difference (RTD) for the full sample of Chilean state agencies analyzed in this study can be found in the Supporting Information section S1 Table.

**Fig 9:**
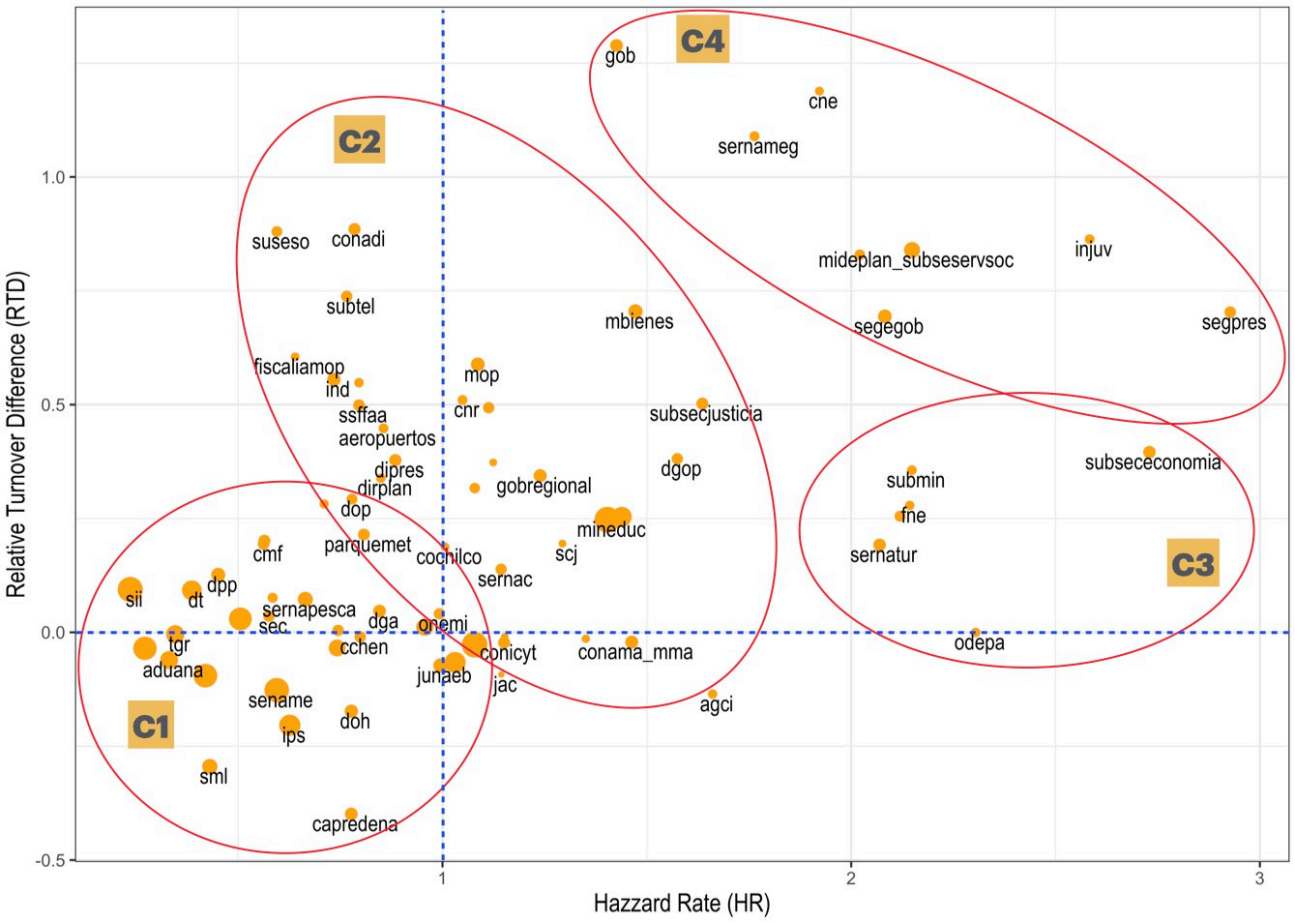
Hazard rate versus relative turnover difference. The figure shows the distribution of Services according to two indices: the Hazard Rate (HR) defined as *f*_*k*_ = exp(*b*_*k*_), where *b*_*k*_ represents frailty, and the Relative Turnover Difference (RTD), defined as $\text{RTD}=\frac{\;\text{Post-electoral}\;\text{Turnover}-\;\text{Regular}\;\text{Turnover}}{\;\text{Regular}\;\text{Turnover}}$. The figure also highlights four groups based on their relative positions according to the values of these indices. The diameter of the circles in the figure is proportional to the size of the agency (Source: authors.).

Using these indices, a hierarchical clustering algorithm based on Euclidean distance and Ward’s method was employed to group agencies according to their employment stability and exposure to political volatility. The clustering revealed significant heterogeneity across agencies, as visualized in Fig 9. The figure illustrates four distinct clusters: the first cluster (C1) consists of agencies with low risk and low turnover, while the second (C2) represents moderate risk and moderate turnover sensitivity to political transitions. The third cluster (C3) includes agencies with high risk but moderate turnover, and the fourth cluster (C4) is characterized by high risk and high turnover, especially during the first year of new administrations.

The clustering results provide valuable insights into how agencies respond to political changes. For example, agencies like the tax authority (SII), the financial regulator (CMF), and the customs authority (Aduana) fall into the most stable cluster (C1), showing low turnover sensitivity to political transitions. These agencies are crucial to government operations and maintain high stability despite electoral cycles. In contrast, agencies closely tied to the presidency and political core (GOB, SEGPRES, SEGEGOB), involved in social policy (MIDEPLAN), or targeting specific demographic groups such as women or youth (SERNAMEG, INJUV) display greater instability and are more vulnerable to electoral shocks, as reflected in cluster C4. Overall, this clustering aligns with the “pockets of effectiveness” framework, highlighting a clear distinction between more and less politicized agencies, linked to how critical their functions are and the policy expertise required in their respective domains.

The two-dimensional map further shows that while job stability measures are correlated, they are not identical. Clusters 2 and 3 reveal agencies where one stability index is high, but the other is low. For example, C2 agencies experience moderate post-electoral shocks but remain relatively stable overall. This cluster includes several undersecretariats (e.g., SUSESO, SUBTEL, SSFFAA, MOP), which have clear political steering roles, explaining the turnover observed in the first year of a new administration. In contrast, C3 agencies are less affected by political shifts but are unstable for perhaps other reasons, such as management practices or workforce characteristics. The National Economic Prosecutor’s Office (FNE), for instance, present in this cluster, is said to have a workforce of young, talented economists who often move on to advanced academic studies or private sector jobs after a few years. Thus, this map underscores the limitations of equating job politicization with instability, as the “pockets of effectiveness” framework tends to do. Although the two may correlate, they are not equivalent, and agencies may experience (in)stability for various reasons and at different times. This underscores the importance of using indices like SHR and RTD to systematically measure both long-term stability and short-term political volatility.

## 5 Discussion

The analysis demonstrates significant variability in agency turnover and staff longevity across Chilean state agencies, which has important implications for understanding both their capacity and autonomy. Staff stability is crucial for capacity, while turnover, especially during post-electoral years, serves as a key indicator of autonomy. Our findings, grounded in the modeling approach, provide answers to the four guiding research questions. First, agency turnover varies markedly between regular and post-electoral years. The unconditional mean model shows that 39% of turnover in regular years is attributable to inter-agency differences, which rises to 65% during post-electoral years. This indicates that political transitions increase turnover heterogeneity. For its part, employment stability also varies starkly by agency: as the Cox models show, a bureaucrat in a state agency with a frailty value one standard deviation above the mean exhibits approximately 67.6% higher risk than civil servants with identical observed covariates working in an average-frailty agency. Importantly, these two metrics, derived from the intraclass correlation coefficient and from frailty values (respectively), are directly comparable across countries. Despite these large differences, overall trends in agency turnover remained stable over time, as shown by the unconditional growth model, which also points to modest convergence in turnover rates among agencies with initially high or low levels of turnover. Interestingly, this stability over time despite three consecutive government turnovers between rival party coalitions counters both the hypothesis that electoral competition would lead to broader employment destabilization, due to the need of politicians for increased bureaucratic responsiveness [60], and also with the hypothesis that it would decrease turnover, due to increased political accountability [61, 62].

Second, turnover and employment stability are only partially explained by observable factors such as contractual regime and the proportion of professionals within an agency. Even after accounting for these covariates in the conditional growth model, 66% of post-electoral turnover variation remains tied to latent agency-specific characteristics. The mixed Cox survival model demonstrated that individual characteristics (age, sex, contract type, rank) and job-related factors explain some of the variation in staff longevity, but only reduce the frailty variance by 19%. Thus, most of the agency heterogeneity remains unexplained. This is consistent with the idea that agencies vary along deeper dimensions such as patronage dynamics and management practices, which are much harder to observe directly.

Third, electoral shocks substantially increase turnover, raising it from 12.5% in regular years to 15.9% in post-electoral years. The results of the mixed linear model after Propensity Score Matching also show that “treated” agency-years have about half a standard deviation higher turnover than “untreated” agency-years. This method, coupled with mediation analysis, further suggests this result is likely to be causal. Political shocks impact agencies unevenly, with some experiencing significantly higher turnover than others, even after controlling for covariates. This indicates a differential response to political transitions, highlighting the varying degrees of autonomy among agencies.

Finally, the clustering analysis based on the Service Hazard Rate (SHR) and Relative Turnover Difference (RTD) illustrates the varying stability of agencies. The mapping of different agencies’ hazard rates and relative turnover differences points to the distinct realities present in the Chilean state. More stable agencies, such as the Internal Revenue Service (SII) and the Financial Regulator (CMF), exhibit low turnover and high stability, even during post-electoral periods. In contrast, politically exposed agencies like SEGEGOB and SEGPRES experience much higher turnover in response to electoral cycles. While the two indicators are correlated, there is significant variation in how agencies map onto each dimension.

Overall, our results support the relevance of a “pockets of effectiveness” framework for studying national bureaucracies even in the context of a moderately “good” country case, whose recruitment is considered mostly meritocratic and with patronage levels somewhat better than the median [41]. We find that Chilean state agencies exhibit substantial heterogeneity in turnover and employment stability, shaped by both observable factors and unobserved characteristics, but in which the explanatory power of the latter is significantly larger. These findings have both methodological and substantive implications.

Methodologically, the finding that observable and potentially important covariates such as agency size, contract regime, or job rank explain only a small proportion of the variance in stability between agencies reinforces the value of models (such as shared frailty models) that can capture the influence of these unobserved factors, demonstrating their relevance to modelling agency-level employment dynamics.

Substantively, these results point to the potentially large influence of patronage and management practices in explaining agency heterogeneity. Regarding political shocks, the high post-electoral turnover in certain agencies—which tend to experience similar patterns across election cycles—suggests they are part of an intertemporal political patronage equilibrium, subject to significant political interference in staffing decisions after elections. This same equilibrium leaves other agencies—such as the Internal Revenue Service—mostly outside the scope of patronage dynamics. In contrast, the differing agency hazard rates, reflecting longer-term trends in stability, are likely influenced by both patronage (which can also occur outside of election years) and factors such as organizational culture or management practices. This is consistent with survey data indicating large differences in human resource management practices across Chilean state agencies; for example, the percentage of recently hired workers reporting recruitment without public advertisement or a rigorous selection process varied from 4% to 70%, depending on the agency [63].

In summary, the empirical application of our methodology to Chilean data has found support for both the literatures on politicized turnover and on “pockets of effectiveness”. Importantly, however, it hasn’t simply registered that there are politically-induced employment shocks and that agencies are diverse, but rather, has provided precise measurements for both these phenomena. This agency characterization and mapping provides a benchmark that is a contribution in its own right to the accumulation of knowledge regarding public administrations in Latin America and beyond, as well as serving as “proof of concept” for the utility of the methodology employed.

## 6 Conclusions

This study developed a replicable methodology for examining staff turnover and longevity across agencies, focusing on both long-term stability and the impacts of political transitions. By leveraging mixed-effects models, Propensity Score Matching (PSM), and survival analysis, we rigorously quantified inter-agency differences and identified patterns of employment stability. The use of SHR and RTD as indices of stability offers a versatile framework that can be applied across various national contexts.

One of the key contributions of this approach is its ability to generate cumulative knowledge about public sector employment. The replicability of the measures and indicators we developed, combined with their applicability to other contexts, allows for the systematic comparison of agency dynamics and heterogeneity across countries, enhancing the comparative study of public administrations. Moreover, by relying on employment tenure data, the methodology offers a straightforward, transparent and highly replicable alternative to more complex and data-heavy approaches, such as factor analysis, for mapping agency heterogeneity.

However, the study has limitations. First, while our models account for observable covariates and unobservable agency effects, we cannot directly test which of these latter factors -such as organizational culture, management practices or patronage dynamics, among others- could further explain variations in turnover and staff longevity. Importantly, at least some of these factors could in principle be observed, if imperfectly, through additional data such as surveys. Second, while this study focused on turnover and longevity, other important determinants of bureaucratic performance, such as public service motivation or the meritocracy of hiring and promotion decisions remain unexplored. These factors are critical for a fuller understanding of bureaucratic performance and should be considered in future studies.

To address both these issues, the combination of large bureaucrat surveys with payroll data within the same national government [22] holds promise to obtain a fuller comprehension of bureaucratic dynamics that incorporates both objective and perception-based information, including on management practices and patronage dynamics within bureaucracies. This route could also help to uncover the relative importance of these two factors as sources of agency variation in employment stability. Indeed, the correlation between the RTD and SHR indices suggests the two may be related, with more politicized agencies perhaps developing more politicized organizational cultures and/or having more unstable management positions, and thus generating poorer management practices than equivalent but less politicized agencies.

In conclusion, this study provides a robust, scalable framework for analyzing employment dynamics in public agencies. It underscores the heterogeneity within the Chilean public sector and offers valuable tools for scholars and policymakers interested in understanding the complex interactions between political transitions, state capacity, and public sector stability. The transparent and replicable methodological approach presented here can be applied across different national contexts, enhancing comparative studies and fostering a deeper understanding of bureaucratic employment dynamics.

## Supporting information

S1 Table Frailty, hazard rate and turnover metrics for state agencies. Service Staffing, Frailties, Hazard Rate (SHR) and Relative Turnover Difference (RTD) for the complete sample of analyzed in this study Chilean State Agencies.
(PDF)

S1 File Additional models. (PDF)
