# Supplementary material for: Mapping employment dynamics in public agencies with payroll data: A methodological framework with an application to Chile
Source: PLoS One. 2024 Dec 31;19(12):e0316386. doi: 10.1371/journal.pone.0316386 (PMC11687733; doi:10.1371/journal.pone.0316386)
Supplement: S1 Table — Service Staffing, Frailties, Hazard Rate (SHR) and Relative Turnover Difference (RTD) for the complete sample of analyzed in this study Chilean State Agencies. (PDF) [file pone.0316386.s001.pdf]

Supporting information

Frailty, Hazard Rate and Turnover metrics for State Agencies

Table 1. Frailty, Hazard Rate and Turnover metrics for State Agencies (Part 1).

Service	Service Full Name	Staffing	Frailty	SHR	MT	PET	RT	RTD
aduana	Customs	1716	-1.1054	0.3311	3.98	3.80	4.05	-0.06
aeropuertos	Airports	184	-0.1559	0.8556	9.20	11.90	8.22	0.45
agci	International Cooperation Agency	145	0.5075	1.6611	18.93	16.98	19.64	-0.14
capredena	National Defense Pension Fund	562	-0.2525	0.7769	8.92	6.00	9.98	-0.40
cchen	Chilean Nuclear Energy Commission	355	-0.2247	0.7987	10.45	10.38	10.48	-0.01
cmf	Financial Market Commission	388	-0.5758	0.5623	6.83	7.75	6.49	0.19
cne	National Energy Commission	111	0.6535	1.9222	18.76	31.18	14.25	1.19
cnr	National Irrigation Commission	186	0.0478	1.0490	13.13	17.45	11.55	0.51
cochilco	Chilean Copper Commission	108	0.0063	1.0063	12.47	14.10	11.87	0.19
conadi	National Indigenous Development Corporation	455	-0.2425	0.7847	9.72	14.82	7.86	0.89
conama_mma	Ministry of the Environment	575	0.3805	1.4630	15.75	15.50	15.84	-0.02
conicyt	National Comm. for Sci. and Tech. Research	423	0.1404	1.1507	13.93	13.70	14.02	-0.02
dcyf	Department of Child and Family Services	355	-0.2949	0.7446	8.00	8.03	7.99	0.00
dga	General Directorate of Water	554	-0.1672	0.8460	7.98	8.25	7.88	0.05
dgop	General Directorate of Public Works	343	0.4540	1.5746	15.47	19.40	14.05	0.38
dicrep	General Directorate of Credit and Collection	344	0.1070	1.1130	12.20	16.10	10.78	0.49
dipres	Budget Office	471	-0.1226	0.8846	13.44	16.82	12.21	0.38
dirplan	National Planning Directorate	160	-0.1634	0.8492	10.45	12.82	9.58	0.34
doh	Hydraulic Works Directorate	646	-0.2525	0.7768	8.13	7.05	8.53	-0.17
dop	Public Works Directorate	291	-0.2504	0.7785	9.15	10.97	8.49	0.29
dpp	Public Prosecutor's Office	778	-0.7955	0.4513	6.49	7.08	6.28	0.13
fiscaliamop	Public Ministry Prosecutor for Public Works	98	-0.4469	0.6396	6.44	8.90	5.55	0.60
fne	National Economic Prosecutor's Office	145	0.7623	2.1432	21.07	25.08	19.61	0.28
gob	Government	558	0.3548	1.4259	14.71	25.05	10.95	1.29
gobregional	Regional Government	602	0.2144	1.2391	17.18	21.15	15.57	0.36

The table displays the random intercepts b_k (frailty), their exponents $f_k = \exp(b_k)$ (Hazard Rates, HR) obtained from a mixed Cox model (frailty model with a Gaussian distribution for the intercepts) for State agencies. HR values greater than one indicate an increased risk for these agencies. Additionally, the table presents Mean Turnover (MT), Post-Electoral Turnover (PET), Regular Turnover (RT), and the Relative Turnover Difference (RTD), defined as $\mathrm{RTD} = \frac{\text{Post-Electoral Turnover} - \text{Regular Turnover}}{\text{Regular Turnover}}$.

Table 2. Frailty, Hazard Rate and Turnover metrics for State Agencies (Part 2).

Service	Service Full Name	Staffing	Frailty	SHR	MT	PET	RT	RTD
hacienda	Ministry of Finance	318	0.7513	2.1197	23.02	27.05	21.55	0.25
inach	Chilean Antarctic Institute	79	0.3006	1.3506	21.55	21.32	21.63	-0.01
ind	National Sports Institute	654	-0.3089	0.7342	7.81	10.58	6.80	0.56
injuv	National Youth Institute	184	0.9491	2.5835	24.41	36.98	19.85	0.86
investchile	InvestChile	45	0.1417	1.1523	14.35	14.25	14.39	-0.01
jac	Civil Aeronautics Board	26	0.1344	1.1439	14.11	13.12	14.46	-0.09
junaeb	National School Aid and Scholarship Board	825	-0.0049	0.9951	13.27	12.55	13.54	-0.07
mbienes	Ministry of National Assets	824	0.3867	1.4721	17.39	24.95	14.64	0.70
mideplan	Ministry of Social Development and Family	1228	0.7650	2.1489	21.77	32.73	17.79	0.84
mop	Ministry of Public Works	742	0.0829	1.0864	12.47	17.12	10.78	0.59
odepa	Office of Agrarian Studies and Policies	149	0.8350	2.3047	19.75	19.75	19.75	0.00
onemi	National Emergency Office	323	-0.0079	0.9922	12.63	13.00	12.49	0.04
parquemet	Metropolitan Park	410	-0.2137	0.8076	10.14	11.65	9.59	0.21
patrimoniocultural	Cultural Heritage Service	1394	-0.2981	0.7422	10.44	10.18	10.54	-0.03
scj	Superintendence of Casinos	56	0.2574	1.2936	16.29	18.50	15.48	0.19
sec	Superintendence of Electricity and Fuels	343	-0.5527	0.5754	8.07	8.28	7.99	0.04
segegob	General Secretariat of Government	725	0.7335	2.0823	19.80	28.30	16.71	0.69
segpres	General Secretariat of the Presidency	372	1.0742	2.9275	25.69	36.85	21.64	0.70
sernac	National Consumer Service	341	0.1340	1.1434	14.64	16.08	14.12	0.14
sernameg	National Women's Service	194	0.5672	1.7633	21.09	34.15	16.35	1.09
sernapesca	National Fisheries and Aquaculture Service	991	-0.4089	0.6644	12.21	12.85	11.98	0.07
sernatur	National Tourism Service	522	0.7273	2.0696	22.62	25.65	21.52	0.19
serviciocivil	Civil Service	157	-0.2285	0.7957	12.49	16.88	10.90	0.55
siss	Superintendence of Sanitary Services	210	-0.5371	0.5845	6.57	6.92	6.44	0.08
sml	Legal Medical Service	1032	-0.8424	0.4307	5.81	4.45	6.31	-0.29

Table 3. Frailty, Hazard Rate and Turnover metrics for State Agencies (Part 3).

Service	Service Full Name	Staffing	Frailty	SHR	MT	PET	RT	RDT
sp	Pensions Supervisor	435	-0.5725	0.5641	6.97	7.95	6.62	0.20
ssffaa	Armed Forces Social Security	386	-0.2285	0.7957	10.87	14.38	9.59	0.50
submin	Ministry of the Interior	175	0.7648	2.1485	22.33	27.65	20.39	0.36
subpesca	Undersecretariat for Fisheries	254	0.0760	1.0789	13.26	16.10	12.23	0.32
subsececonomia	Undersecretariat of Economy	509	1.0042	2.7297	26.73	33.75	24.18	0.40
subsecjusticia	Undersecretariat of Justice	440	0.4921	1.6358	19.40	25.70	17.11	0.50
subtel	Undersecretariat of Telecommunications	344	-0.2670	0.7657	10.64	15.45	8.89	0.74
subtrab	Undersecretariat of Labor	258	0.7037	2.0212	21.45	32.12	17.56	0.83
subtrans	Undersecretariat of Transport	1495	-0.0443	0.9567	11.38	11.47	11.35	0.01
superir	Superintendence of Insolvency	133	-0.3420	0.7103	11.55	13.78	10.75	0.28
suseso	Social Security Superintendence	281	-0.5194	0.5949	7.49	11.40	6.06	0.88
tgr	Treasury	1821	-1.0610	0.3461	4.21	4.20	4.22	0.00
uaf	Financial Analysis Unit	54	0.1170	1.1241	14.17	17.70	12.89	0.37
dgac	General Directorate of Civil Aeronautics	3695	-1.3022	0.2719	3.08	3.00	3.11	-0.04
dt	Labor Department	2268	-0.9502	0.3866	4.03	4.30	3.94	0.09
ine	National Statistics Institute	4135	1.3586	3.8906	34.01	19.32	39.35	-0.51
ips	Social Security Institute	2818	-0.4678	0.6264	6.92	5.82	7.32	-0.20
mineduc	Ministry of Education	4735	0.3389	1.4034	20.27	23.73	19.02	0.25
registrocivil	Civil Registry and Identification Service	3430	-0.6824	0.5054	7.07	7.22	7.02	0.03
sag	Agricultural and Livestock Service	4691	0.0757	1.0786	15.61	15.30	15.73	-0.03
sename	National Service for Minors	4176	-0.5202	0.5944	7.55	6.82	7.82	-0.13
servius	Housing and Urbanism Services	2758	0.0304	1.0309	11.89	11.30	12.10	-0.07
sii	Internal Revenue Service	4471	-1.4440	0.2360	2.60	2.78	2.54	0.09
subsecminvu	Undersecretariat of Housing and Urbanism	2308	0.3639	1.4389	16.86	19.80	15.79	0.25
vialidad	Roads Directorate	3795	-0.8677	0.4199	4.12	4.40	4.04	0.09
