# Supplementary material for: Mapping employment dynamics in public agencies with payroll data: A methodological framework with an application to Chile
Source: PLoS One. 2024 Dec 31;19(12):e0316386. doi: 10.1371/journal.pone.0316386 (PMC11687733; doi:10.1371/journal.pone.0316386)
Supplement: S1 File — (PDF) [file pone.0316386.s002.pdf]

1 Supporting information

1.1 About the Data.

The dataset underwent extensive processing due to its dispersed and inconsistent initial format. The data was sourced through web scraping from the Transparency Portal [1], requiring individual tracking of agencies before 2017/2018. This process often involved downloading multiple files per agency each month, segmented by employment regime. After 2018, data centralization in the Transparency Council website simplified access but introduced temporary gaps. Following download, the data was meticulously cleaned, addressing issues like mixed columns, inconsistent date formats, and varied character encoding. Name matching and record linkage were particularly challenging due to variations in naming conventions, which were resolved through fuzzy matching algorithms and linkage to the national electoral roll, achieving 90% accuracy in unique identification.

To prepare for analysis, the dataset was standardized to monthly records, even for agencies initially reporting yearly. Job spells were determined by tracking individual appearances across monthly data, accounting for partial data gaps, with a 13-month gap treated as a job spell end. Data validity was ensured by eliminating inconsistencies, such as entries where start and exit dates conflicted with actual appearance in the dataset. Decision rules were applied to assign each individual to a single agency per month, thus avoiding duplication and improving dataset reliability. This cleaning and validation process yielded a final dataset with 15.8 million records, ready for rigorous analysis of employment dynamics across public agencies.

The dataset includes information from over 200 agencies across 24 ministries, encompassing regional and provincial delegations, regional housing services, and all line ministries and their respective agencies (except the health sector), and spans over 15 years (2006 – 2020).

We validated our dataset using two Budget Office (DIPRES) sources from 2011 and 2020. For 2006-2010, we matched 112 agencies, focusing on permanent and yearly contracts, as DIPRES reports only minimal data for temporary employees. From 2011-2019, we expanded to 137 agencies using DIPRES's published data, again centering on core staff. Although our dataset includes slightly more temporary staff on average, it aligns closely with DIPRES data. We excluded employees under private sector labor laws reported by DIPRES since 2011, as they represent a marginal category. For coverage assessment, we calculated missing data by excluding agency/years where both our dataset and DIPRES report zero employees. Between 2006-2010, about 15% of agency/years had missing data, which improved to 7% for 2011-2019, reflecting an overall trend of increased data completeness over time.

To assess data completeness, we evaluated the alignment between our dataset and DIPRES data for each agency-year. Agency-years were classified as "Good" if the deviation from DIPRES data was within ±10%, "Some problems" for 10-30%, "Poor" for 30-50%, and "Very poor" if over 50%. Results indicate that most agency-years fall into the "Good" category, with 84.9% for 2006-2010 and 88.7% for 2011-2019, demonstrating strong correspondence. This match suggests reliable reporting by services and highlights the importance of our name-matching procedure, which prevented about 30% spurious entries. Deviations are primarily due to missing data in specific large services (e.g., prison guards and nursery services).

This effort was part of a research and development project funded by the National Research and Development Agency (ANID) through the FONDEF IDeA I+D 2019 Project (ID19I10198), titled "*Towards State Modernization: Analytical Data-driven Modeling of the Chilean State's Capacity and Autonomy*".

Fig. 1 shows a typical snippet of the data for an agency (corresponding to the

beginning of the list of yearly contract workers for January 2018).

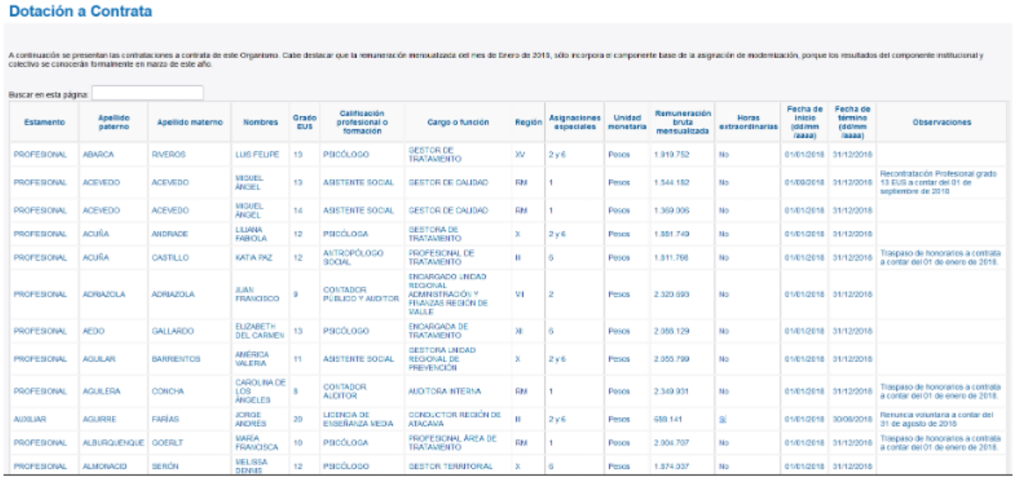

Dotación a Contrata

A continuación se presentan las contrataciones a contrata de este Organismo. Cabe destacar que la remuneración mensualizada del mes de Enero de 2018, sólo incorpora el componente base de la asignación de modernización, porque los resultados del componente institucional y colectivo se conocerán formalmente en marzo de este año.

Buscar en esta página:

Estamento	Apellido paterno	Apellido materno	Nombres	Grado EUS	Calificación profesional o formación	Cargo o función	Región	Asignaciones especiales	Unidad monetaria	Remuneración bruta mensualizada	Horas extraordinarias	Fecha de inicio (dd/mm /aaaa)	Fecha de término (dd/mm /aaaa)	Observaciones
PROFESIONAL	ABARCA	RIVEROS	LUIS FELIPE	13	PSICÓLOGO	GESTOR DE TRATAMIENTO	XV	2 y 6	Pesos	1.919.752	No	01/01/2018	31/12/2018	
PROFESIONAL	ACEVEDO	ACEVEDO	MIGUEL ÁNGEL	13	ASISTENTE SOCIAL	GESTOR DE CALIDAD	RM	1	Pesos	1.544.182	No	01/09/2018	31/12/2018	Recontratación Profesional grado 13 EUS a contar del 01 de septiembre de 2018
PROFESIONAL	ACEVEDO	ACEVEDO	MIGUEL ÁNGEL	14	ASISTENTE SOCIAL	GESTOR DE CALIDAD	RM	1	Pesos	1.369.006	No	01/01/2018	31/12/2018	
PROFESIONAL	ACUÑA	ANDRADE	LILIANA FABIOLA	12	PSICÓLOGA	GESTORA DE TRATAMIENTO	X	2 y 6	Pesos	1.881.749	No	01/01/2018	31/12/2018	
PROFESIONAL	ACUÑA	CASTILLO	KATIA PAZ	12	ANTROPÓLOGO SOCIAL	PROFESIONAL DE TRATAMIENTO	II	6	Pesos	1.811.798	No	01/01/2018	31/12/2018	Traspaso de honorarios a contrata a contar del 01 de enero de 2018.
PROFESIONAL	ADRIAZOLA	ADRIAZOLA	JUAN FRANCISCO	9	CONTADOR PÚBLICO Y AUDITOR	ENCARGADO UNIDAD REGIONAL ADMINISTRACIÓN Y FINANZAS REGIÓN DE VALLE	VI	2	Pesos	2.320.693	No	01/01/2018	31/12/2018	
PROFESIONAL	AEDO	GALLARDO	ELIZABETH DEL CARMEN	13	PSICÓLOGO	ENCARGADA DE TRATAMIENTO	XI	6	Pesos	2.088.129	No	01/01/2018	31/12/2018	
PROFESIONAL	AGUILAR	BARRIENTOS	AMÉRICA VALERIA	11	ASISTENTE SOCIAL	GESTORA UNIDAD REGIONAL DE PREVENCIÓN	X	2 y 6	Pesos	2.055.799	No	01/01/2018	31/12/2018	
PROFESIONAL	AGUILERA	CONCHA	CAROLINA DE LOS ÁNGELES	8	CONTADOR AUDITOR	AUDITORA INTERNA	RM	1	Pesos	2.349.931	No	01/01/2018	31/12/2018	Traspaso de honorarios a contrata a contar del 01 de enero de 2018.
AUXILIAR	AGUIRRE	FARÍAS	JORGE ANDRÉS	20	LICENCIA DE ENSEÑANZA MEDIA	CONDUCTOR REGIÓN DE ATACAMA	III	2 y 6	Pesos	588.141	Sí	01/01/2018	30/08/2018	Renuncia voluntaria a contar del 31 de agosto de 2018
PROFESIONAL	ALBURQUENQUE	GOERLT	MARÍA FRANCISCA	10	PSICÓLOGA	PROFESIONAL ÁREA DE TRATAMIENTO	RM	1	Pesos	2.004.707	No	01/01/2018	31/12/2018	Traspaso de honorarios a contrata a contar del 01 de enero de 2018.
PROFESIONAL	ALMONACID	SERÓN	MELISSA DENNIS	12	PSICÓLOGO	GESTOR TERRITORIAL	X	6	Pesos	1.874.037	No	01/01/2018	31/12/2018	

Fig 1. Transparency Portal Data. Example of data that was webscraped.

The raw data downloaded from the Transparency Portal contains the following variables:

- Year
- Month
- Rank
- Full name
- Position or function
- EUS grade or workday
- Professional qualification or training
- Region
- Special allowances
- Gross monthly remuneration
- Net monthly remuneration
- Additional remuneration
- Incentive bonus remuneration
- Right to overtime
- Daytime overtime amounts and hours
- Nighttime overtime amounts and hours
- Holiday overtime amounts and hours
- Start date (dd/mm/yy)
- End date (dd/mm/yy)

- Travel expenses
- Observations

The curated datasets utilized specifically for this analysis can be downloaded from **[2]**.

Service short names and full names in Spanish and English can also be downloaded from **[2]**.

The variables contained in these data are given in the table 1:

**Variable**	**Description**
`ID`	Unique identifier for the employee
`Spell`	The spell is an indicator of the streak or continuous period of time in service, 1 first streak, 2 second, etc.
`Grade`	Grade level of the employee; lower values indicate higher ranks
`year`	Year
`Month`	Month
`Status`	Employment status: 0 if the employee leaves in that month-year, 1 if still active
`dateIn`	Date of entry into the service
`dateOut`	Date of exit from the service
`Longevity`	Employee's longevity in the service
`time_start`	Observation start time for the employee
`time_end`	Observation end time for the employee
`Service`	Short name of the service
`Sex`	Gender of the employee
`ContractRegime`	Employment regime (e.g., "Temporary", "Contractual", "Permanent")
`Rank`	Job rank (e.g., "No information", "Professional", "Assistant", "Administrative", "Technician", "Managerial", "Inspector", "Government Authority")
`Label`	Month and year label
`year_month`	Continuous numerical format for month and year
`Label1`	Combined label for service, month, and year
`staffing`	Monthly staffing level of the service
`turnover`	Monthly turnover rate of the service
`meanturnover`	Annual mean turnover rate
`churns`	Number of employees leaving the service in the given month

Table 1. Variable Names and Descriptions

References

[1] **https://www.portaltransparencia.cl/PortalPdT/**

[2] **Harvard Dataverse. https://doi.org/10.7910/DVN/Z2EPOS.**

1.2 The unconditional growth model with quadratic effect of time.

While modeling linear trends over time is often a good approximation, we can also consider modeling the quadratic effect of time by adding terms for both time and the square of time. In this case:

- $\hat{\alpha}_0 = 13.95$: The mean staff turnover for all agencies in 2006.
- $\hat{\beta}_0 = 1.05$: The rate of change in staff turnover for the Year.
- $\hat{\beta}_1 = -0.078$: The rate of change in staff turnover for Year^2.
- $\hat{\sigma}^2 = 32.76$: The staff turnover variance within-agency deviations.
- $\hat{\sigma}_u^2 = 65.49$: The staff turnover variance between agencies in 2006.

1.2.1 Interpretation of Results

Random Effects:

Service (Intercept): The variance of the intercept at the service level is 65.49, with a standard deviation of 8.093. This suggests significant variation in turnover across different services.

Residual: The residual variance, representing variation within services, is 32.76 with a standard deviation of 5.724.

The fact that the between-service variance (65.49) is greater than the within-service variance (32.76) indicates that turnover varies more between services than within them. This reinforces the idea that there are significant differences across services in terms of turnover.

Fixed Effects:

The fixed effects represent the estimated coefficients for *Year*, *Year2*, and the intercept.

Intercept:

- Estimate: 13.95
- This indicates that when *Year* and *Year2* are 0 (the baseline of the model), the expected turnover is 13.95 units.
- Standard Error: 1.219
- The low standard error indicates a precise estimate of the intercept.
- t-value: 11.445, $p < 2e - 16$
- The intercept is highly significant ($p < 0.001$), meaning that the baseline level of turnover, without the effects of *Year* and *Year2*, is significantly different from 0.

Year (Linear Term):

- Estimate: 1.05
- Each additional year is associated with an *increase of 1.05 units in turnover.* This suggests a positive trend in turnover over time.
- Standard Error: 0.278
- The low standard error indicates precise estimation.
- t-value: 3.78, p = 0.000209
- The *Year* variable is highly significant ($p < 0.001$), meaning that time has an important impact on turnover.

Year2 (Quadratic Term):

- Estimate: -0.078
- The negative coefficient indicates a *downward curvature in the relationship between time and turnover.* While turnover increases over time (based on the linear *Year* term), the *rate of increase slows down* due to this quadratic term.
- Standard Error: 0.022
- The low standard error suggests that this estimate is also precise.
- t-value: -3.516, p = 0.000546
- *Year2* is also highly significant (p ¡ 0.001), confirming the non-linear relationship between time and turnover.

Correlation of Fixed Effects:

- **Year and Year2**: The correlation between *Year* and *Year2* is -0.958, indicating a high negative correlation, which is expected since *Year2* is a quadratic function of time and is intrinsically related to *Year*.
- The negative correlation between the *Intercept* and *Year* is moderate, meaning that the baseline level of turnover and its time trend are somewhat related.

General Summary:
This model captures how turnover in Chilean state services varies over time, with significant variation between services.

Temporal Trend Interpretation: Turnover tends to increase over time, but at a decreasing rate, as indicated by the negative quadratic term.

Service-Level Heterogeneity: The significant between-service variance suggests that some services experience much higher turnover than others.

Importance of Random Effects: The inclusion of random effects for services allows us to capture unobserved differences between services, adding complexity and realism to the model.

This analysis suggests that while turnover generally increases with time, individual services follow distinct patterns, with some being more exposed to turnover than others.

1.3 The Conditional Growth Models for professional/non-professional agencies.

This "intermediate" model includes only the binary variable "Professional", where $\text{Professional}_i = 1$ indicates that agency i is classified as a "professional agency" (annual average professional density exceeds the median $Q_2^{(P)}$, calculated from the distribution of the proportion of professionals across all agencies for each year). In contrast, $\text{Professional}_i = 0$ designates a "non-professional agency", where the annual average professional density is less than or equal to the median.

$$
\begin{aligned}
a_i &= \alpha_0 + \alpha_1 \text{Professional}_i + u_i \\
b_i &= \beta_0 + \beta_1 \text{Professional}_i + v_i
\end{aligned}
$$

Where $\text{Professional}_i = 1$ if agency i is a "professional agency" (annual professional density is greater than Q_2), and $\text{Professional}_i = 0$ if otherwise. In addition, the error terms at level two are assumed to follow a multivariate normal distribution.

Using a binary predictor at level two, such as agency professional status (as an example of variables that characterize the agency), we can define our Level Two Model to differentiate between non–professional and professional agencies.

For non–professional agencies:

$$\begin{aligned} a_i &= \alpha_0 + u_i \\ b_i &= \beta_0 + v_i, \end{aligned}$$

For professional agencies:

$$\begin{aligned} a_i &= (\alpha_0 + \alpha_1) + u_i \\ b_i &= (\beta_0 + \beta_1) + v_i \end{aligned}$$

Writing the level two model in this manner helps us interpret the model parameters from our two-level model. We use statistical software (the $lmer()$ function from the **lme4** package in R) to obtain parameter estimates using our data, after first converting our level one and level two models into a composite model with fixed effects and random effects separated:

$$\begin{aligned} Y_{ij} &= a_i + b_i \text{Year}_{ij} + \epsilon_{ij} \\ &= (\alpha_0 + \alpha_1 \text{Professional}_i + u_i) + (\beta_0 + \beta_1 \text{Professional}_i + v_i)\text{Year}_{ij} + \epsilon_{ij} \\ &= [\alpha_0 + \beta_0 \text{Year}_{ij} + \alpha_1 \text{Professional}_i + \beta_1 \text{Professional}_i \times \text{Year}_{ij}] + \\ &\quad [u_i + v_i \text{Year}_{ij} + \epsilon_{ij}] \end{aligned} \quad (1)$$

The results from fitting the Conditional Growth Model 1, presented in Table 2, provide insights into the dynamics of staff turnover within Chilean state agencies, particularly highlighting the differences between professional and non-professional agencies.

Table 2. Summary of Conditional Growth Model with professional/non-professional agencies.

Parameter	Estimate	Description
$\hat{\alpha}_0$ **(Intercept)**	13.82	Mean staff turnover for non-professional agencies in 2006
$\hat{\alpha}_1$ **(Professional)**	2.59	Higher turnover in professional agencies (non-significant)
$\hat{\beta}_0$ **(Year effect)**	0.51	Annual increase in turnover for non-professional agencies
$\hat{\beta}_1$ **(Year × Professional)**	-0.60	Yearly decrease in turnover for professional agencies
**Random Effects**	**Variance**	**Description**
$\hat{\sigma}_u$ **(2006 turnover variance)**	8.81	Standard deviation of turnover between agencies after controlling for professional status
$\hat{\sigma}_v$ **(Yearly change variance)**	0.49	Standard deviation of yearly turnover change across electoral years
$\hat{\rho}_{uv}$ **(Correlation)**	-0.40	Correlation between 2006 turnover and yearly change
$\hat{\sigma}$ **(Residual SD)**	5.17	Standard deviation of residual turnover trends
**Model Fit**		
**Conditional** R^2	0.73	Overall explanatory power of the model
**Marginal** R^2	0.02	Variance explained by fixed effects

The fixed effects estimates provide information on the average turnover rates in 2006 and how these rates evolve over time. Specifically, the mean staff turnover for

non-professional agencies in 2006 was estimated at 13.82%. In contrast, professional agencies exhibited an average turnover rate that was 2.59 percentage points higher than non-professional agencies in the same year, although this difference lacks statistical significance, indicating that the observed higher turnover in professional agencies may not be a consistent trend across all agencies.

Further, the analysis shows that non-professional agencies experience an average yearly increase in turnover of approximately 0.51%. On the other hand, professional agencies show a different trend, with an estimated average decrease of 0.60% in turnover during each successive first year of a new government. This suggests that, while non-professional agencies face increasing turnover over time, professional agencies may be better able to stabilize their staff, particularly in the context of political transitions.

The random effects or variance components provide further detail on the variability of turnover between and within agencies. The standard deviation of turnover across agencies in 2006, after accounting for professional status, is estimated at 8.81, indicating considerable variability in turnover rates across agencies. Additionally, the standard deviation for yearly changes in turnover during the four post-electoral years is estimated at 0.49, showing moderate variability in how turnover rates evolve during electoral transitions. The correlation between turnover in 2006 and subsequent yearly changes in turnover is negative (-0.40), suggesting that agencies with higher initial turnover rates in 2006 tend to experience smaller increases (or even decreases) in turnover over time, and vice versa.

Finally, the residual standard deviation, which captures the variability in turnover trends that is not explained by the fixed effects, is estimated at 5.17. This indicates some residual variability in turnover rates that is not fully accounted for by the factors included in the model, such as professional status or time.

Overall, the model exhibits a strong fit, with a conditional R^2 of 0.73, indicating that the combination of fixed and random effects explains 73% of the total variance in turnover rates. However, the marginal R^2, which reflects the explanatory power of the fixed effects alone, is only 0.02, suggesting that much of the variation is driven by unobserved heterogeneity between agencies, which is captured by the random effects. This highlights the importance of accounting for agency-specific factors when analyzing turnover dynamics.

1.4 Modeling Civil Servant Turnover: Impact of Temporary Contracts and Professional Proportions During Government Transitions.

A model with proportion of civil servants on temporary contracts and proportion of professionals, as continuous variables, for the years of installation of new administrations.

$$Y_{ij} = \beta_0 + \beta_1 \text{Year}_j + \beta_2 \text{Prop\_professional}_{ij} + \beta_3 \text{Prop\_temporary}_{ij} \quad (2)$$
$$+u_{0i} + u_{1i} \text{Year}_j + \epsilon_{ij} \quad (3)$$

Where

- Y_{ij}: Represents the turnover rate for service i in year j.
- β_0: The global intercept, representing the baseline turnover rate across all services when other covariates are held constant.
- β_1: The fixed effect coefficient for the variable *Year*. This captures the influence of the specific year on the turnover rate.

- β_2: The fixed effect coefficient for the variable *Prop_professional*. This represents the effect of the proportion of professional staff in a service on turnover.

- β_3: The fixed effect coefficient for the variable *Prop_temporary*. This quantifies how the proportion of temporary staff in a service affects turnover.

- u_{0i}: The random intercept for service i, representing the service-specific deviation from the global intercept. It captures unobserved heterogeneity between services.

- u_{1i}: The random slope for the *Year* variable for each service i, capturing how the effect of the year on turnover varies across services.

- ϵ_{ij}: The residual error term, representing the unexplained variability in turnover within each service over time, assumed to follow a normal distribution with mean 0 and variance $\sigma^2_{\text{Residual}}$.

Variance Components:

- $\sigma^2_{\text{Service}}$: The variance between services, indicating how much turnover rates differ from one service to another.

- σ^2_{Year}: The variance of the year-specific effects within each service, showing how the effect of time on turnover differs across services.

- $\sigma^2_{\text{Residual}}$: The residual variance, representing the variability within services not explained by the model.

This mixed model allows us to account for both fixed effects (overall trends across all services) and random effects (service-specific deviations), helping to better understand the variability in turnover rates over time and across different services.

Model fitting results:

**Fixed Effects**	**Estimate**	**Std. Error**	**t value**	**Pr($>\|t\|$)**
(Intercept)	3.116	1.512	2.062	0.0407 *
Year	0.058	0.085	0.686	0.4944
Prop_professional	0.100	0.029	3.474	0.0006 ***
Prop_temporary	0.258	0.021	12.085	$< 2e-16$ ***
**Random Effects**	**Variance**	**Std. Dev.**	**Correlation**	
Intercept (Service)	30.083	5.485	-	
Year (Service)	0.061	0.248	-0.04	
Residual	22.090	4.700	-	

Table 3. Results from the Linear Mixed Model on Turnover during Government Change Periods

The linear mixed model (LMM) was fitted to the annual turnover data, specifically for the periods of government change. This model includes Year, Proportion of Professional Staff (Prop_professional), and Proportion of Temporary Staff (Prop_temporary) as fixed effects, and allows for random intercepts and slopes for the variable Year across different services. Here's a detailed interpretation of the results:

Fixed Effects:

- (Intercept): The estimated intercept of 3.116 (p-value = 0.040) suggests that, when all other covariates (Year, Proportion of Professional Staff, and Proportion of Temporary Staff) are at their reference levels, the expected turnover is approximately 3.12. This result is statistically significant.

- Year: The coefficient for Year (0.058, p-value = 0.494) is not statistically significant, implying that the overall trend of turnover across years does not change in a meaningful way during the periods of government transition in this dataset.
- Prop_professional: The proportion of professional staff has a positive and significant effect on turnover (estimate = 0.100, p-value < 0.001). This indicates that agencies with a higher proportion of professional staff experience higher turnover during government change periods.
- Prop_temporary: The proportion of temporary staff also has a positive and highly significant effect on turnover (estimate = 0.258, p-value < 0.001). Agencies with a higher proportion of temporary staff tend to experience even higher turnover during these periods, highlighting the instability associated with temporary contracts.

Random Effects:

- Intercept Variance (Service): The variance of the intercept across services is estimated at 30.08, with a standard deviation of 5.48. This suggests considerable variation in baseline turnover between services.
- Slope for Year (Service): The variance for the slope of Year across services is relatively small at 0.061 (standard deviation = 0.247), indicating little variability in how turnover trends over time across different services. The correlation between the random intercept and random slope is small (-0.04), suggesting little to no relationship between a service's baseline turnover and how turnover changes with Year.
- Residual Variance: The residual variance (within-service variability) is estimated at 22.09 (standard deviation = 4.70), indicating a substantial amount of variability in turnover that is not explained by the fixed effects or the service-level random effects.
- Model Convergence: The model has converged with a note indicating that the maximum gradient (0.00273) was slightly above the tolerance level (0.002). This could suggest that the optimization algorithm had some difficulty, but overall the model results are reliable for interpretation.

The lack of a significant effect for Year suggests that, over time, the turnover rates in the periods of government change do not exhibit a strong trend. The significant positive coefficients for Prop_professional and Prop_temporary indicate that agencies with higher proportions of professional and temporary staff experience greater turnover during periods of government transition. This suggests that organizational structure, particularly staffing composition, plays a key role in how agencies respond to political shifts.

To further analyze the variability in turnover between services and within services, we calculate the Intraclass Correlation Coefficient (ICC). The ICC quantifies how much of the total variance is attributable to differences between services (i.e., the random intercept variance) versus within services (i.e., the residual variance). The ICC can be interpreted as the proportion of total variance that is due to differences between services.

$$\text{ICC} = \frac{\sigma^2_{\text{Service}}}{\sigma^2_{\text{Service}} + \sigma^2_{\text{Residual}}}$$

Where:

- $\sigma^2_{\text{Service}}$ is the variance of the random intercept for services.
- $\sigma^2_{\text{Residual}}$ is the residual variance (within-service variability).

From the model results, we have:

- $\sigma^2_{\text{Service}} = 30.08353$ (the variance between services)
- $\sigma^2_{\text{Residual}} = 22.09031$ (the residual variance within services)

Now we can calculate the ICC:

$$\text{ICC} = \frac{30.08353}{30.08353 + 22.09031} = \frac{30.08353}{52.17384} \approx 0.577$$

The ICC of 0.577 means that approximately 57.7% of the total variance in turnover is attributable to differences between services, while the remaining 42.3% of the variance is due to within-service differences (i.e., differences within services across different time points or unobserved factors within each service). it is clear that organizational factors unique to each service (such as management practices, service-specific policies, or other contextual factors) play a significant role in determining turnover rates.

The residual variance of 22.09031 (standard deviation of 4.700) represents the variability in turnover within individual services, across different years or due to unobserved factors. This suggests that there are still important dynamics occurring within services that are not fully captured by the fixed effects or the random service effect. For example, internal organizational changes, temporary management decisions, or other context-specific events could be driving the residual variance.

The fact that more than half of the total variance is attributed to between-service differences suggests that turnover is heavily influenced by service-specific factors. These could include: Different hiring and retention policies across services, Different levels of exposure to political shifts or external shocks, Varied levels of professionalization or temporary contract usage in different services.

On the other hand, the substantial within-service variance implies that there are fluctuations in turnover within services that cannot be explained solely by service-level characteristics. These fluctuations might reflect time-sensitive factors such as short-term policies or external events affecting particular services at different times.

1.5 Mediation Model and Sensitivity Analysis Results

The mediation analysis results indicate that the proportion of temporary employees (prop_temporary) acts as a significant mediator between government change (treatment) and turnover (avg_turnover). The Average Causal Mediation Effect (ACME) is negative and statistically significant, suggesting that services with a higher proportion of temporary workers already experience high turnover due to the inherent instability of their employment. As a result, the additional effect of government change on turnover is smaller in these services. On the other hand, the Average Direct Effect (ADE) is positive and significant, indicating that the direct effect of government change on turnover remains substantial. The total effect is also positive, confirming that government transitions lead to higher turnover, but the presence of temporary workers mediates part of this effect. The proportion of mediated effect is around -18%, meaning that the mediating role of temporary employees somewhat offsets the overall impact of government change on turnover. We use R package **mediation**:

```
library(mediation)
#  Fit the mediation model
```

```
# Model 1: Effect of treatment on the proportion of temporary employees
mediator_model <- lm(prop_temporary ~ treatment +
staffing + prop_professional, data = df)

# Model 2: Effect of treatment and mediator (proportion of temporary employees)
outcome_model <- lm(avg_turnover ~ treatment +
prop_temporary + staffing + prop_professional, data = df)

# Run the mediation analysis

med_analysis <- mediate(mediator_model, outcome_model, treat = "treatment",
 mediator = "prop_temporary", boot = TRUE, sims = 1000)

# View the results
summary(med_analysis)
```

Table 4. Causal Mediation Analysis Results

	Estimate	95% CI Lower	95% CI Upper	p-value
ACME	-0.0915	-0.1327	-0.0500	$< 2 \times 10^{-16}$
ADE	0.5981	0.4004	0.8200	$< 2 \times 10^{-16}$
Total Effect	0.5065	0.2986	0.7400	$< 2 \times 10^{-16}$
Prop. Mediated	-0.1807	-0.3659	-0.0900	$< 2 \times 10^{-16}$

And the sensitivity analysis:

```
sens.cont <- medsens(med_analysis, rho.by = 0.05)
summary(sens.cont)
```

Produce:

Table 5. Mediation Sensitivity Analysis for Average Causal Mediation Effect

**Rho**	**ACME**	**95% CI Lower**	**95% CI Upper**	**R^2_M*R^2_Y***	**R^2_M R^2_Y**
0.2	-0.0059	-0.02	0.0083	0.04	0.0228

Rho at which ACME = 0: 0.2

R^2_M*R^2_Y* at which ACME = 0: 0.04
R^2_M R^2_Y at which ACME = 0: 0.0228

The results from the sensitivity analysis using the medsens function indicate how sensitive the estimated Average Causal Mediation Effect (ACME) is to the potential presence of unmeasured confounding in both the mediator-outcome relationship and the treatment-mediator relationship.

Despite the presence of some mediating effects, the direct effect of government change (ADE) remains significant, as seen in the results. This suggests that while part of the turnover effect can be explained by the proportion of temporary workers, the government change itself has a strong and direct influence on staff turnover, independent of this mediator.

1.5.1 Propensity Score Matching results.

To ensure comparability between the treated and control groups, we applied propensity score matching using the nearest neighbor method.

Before Matching, there was a notable imbalance between groups. For instance, the standardized mean difference for Prop_temporary was -0.3555, indicating a significantly lower proportion of temporary staff in the treated group. The variance ratios and eCDF metrics also showed considerable discrepancies.

After Matching, the standardized mean differences were reduced to near zero (e.g., Prop_temporary -0.0084), and variance ratios improved, indicating strong balance between the groups. The eCDF Mean dropped from 0.0943 to 0.0008, further confirming distributional alignment.

These results confirm that the propensity score matching effectively balanced key covariates, allowing for a more reliable comparison between the groups.

1.5.2 Results of the Mixed Linear Model applied to the matched data

Table 6. Results of the Mixed Linear Model on Matched Data: Turnover Analysis with Fixed and Random Effects.

**Fixed Effects**	**Estimate**	**Std. Error**	**t value**	**Pr(> |t|)**
(Intercept)	1.321	0.089	14.829	0.00
treatment	0.507	0.109	4.659	0.00
staffing_scaled	0.044	0.082	0.535	0.59468
Prop_temporary_scaled	1.019	0.122	8.382	0.00
Prop_professional_scaled	0.054	0.090	0.603	0.54887
Prop_contractual_scaled	0.121	0.112	1.073	0.28754
Prop_male_scaled	0.011	0.078	0.139	0.88971
Prop_managerial_scaled	0.200	0.077	2.605	0.00993
Prop_administrative_scaled	0.089	0.083	1.072	0.28905
**Random Effects**	**Variance**	**Std.Dev.**		
Service (Intercept)	0.1313	0.3624		
Residual	5.2968	2.3015		

The results of the mixed linear model applied to the matched data, obtained through propensity score matching (see Table 6), provide valuable insights into the factors driving staff turnover in state agencies. To ensure comparability across variables, all covariates were scaled before fitting the model.

The intercept ($\beta_0 = 1.321$) is highly significant ($p < 10^{-16}$), indicating a notable baseline turnover rate across services, which reflects the average turnover rate in the absence of other covariates.

The treatment variable, which represents a change in government, has a statistically significant positive effect on turnover ($\beta = 0.507$, $p = 3.41 \times 10^{-6}$). This suggests that government transitions are associated with a measurable increase in staff turnover, confirming the disruption often caused by political shifts.

Among the covariates, the proportion of temporary staff (*Prop_temporary_scaled*) has the strongest and most significant effect on turnover ($\beta = 1.019$, $p = 3.10 \times 10^{-12}$).

Other covariates, such as *staffing*, *proportion of professionals*, *proportion of contractual officials*, and *proportion of male employees*, were not statistically significant, indicating that their influence on turnover may be limited in this context. However, the *proportion of managerial staff* (*Prop_managerial_scaled*) has a statistically significant positive effect on turnover ($\beta = 0.200$, $p = 0.00993$). This suggests that services with a higher percentage of managerial staff experience slightly higher turnover rates during the period analyzed, potentially due to the political sensitivity of managerial roles.

In summary, the model highlights the importance of both organizational characteristics and service-specific factors in driving turnover, with government transitions and the proportion of temporary staff emerging as the most influential

variables. Nonetheless, the significant within-service variance suggests that additional factors, perhaps related to organizational culture or political context, could further explain turnover fluctuations during the observed period.

1.5.3 Mediation analysis.

In order to better understand the mechanisms driving the relationship between government transitions and staff turnover, we employed mediation analysis. This approach allows us to examine not just the direct effect of government transitions on turnover, but also the indirect pathways through which this influence may be exerted. Specifically, we focus on the proportion of temporary contracts as a mediating variable. Temporary contracts are known to introduce volatility in public sector employment due to their inherent instability and susceptibility to political changes, making them a natural candidate for mediation. By analyzing this variable, we can disentangle the direct impact of political transitions from the indirect effects mediated through shifts in workforce composition.

The mediation analysis results reveal that the proportion of temporary contracts plays a significant mediating role in the relationship between government transitions and turnover. The Average Causal Mediation Effect (ACME) is estimated at -0.111, with a 95% confidence interval ranging from -0.148 to -0.08, indicating that the mediation effect is both negative and statistically significant. The Average Direct Effect (ADE) is 0.630, meaning that even after accounting for the mediating role of temporary contracts, government transitions still have a direct and significant positive impact on turnover. The total effect of government transitions on turnover is estimated at 0.519, reinforcing that the combination of direct and mediated effects leads to an increase in turnover following political changes.

The proportion mediated is -0.214, meaning that approximately 21.4% of the total effect of government transitions on turnover is explained through the mediating influence of temporary contract proportions. This highlights the critical role that workforce composition plays in turnover dynamics during political transitions. These findings imply that while temporary contracts amplify political vulnerability by mediating a portion of the turnover effect, the direct impact of political changes remains substantial.

The metrics provide robust evidence supporting the significance of the mediation effect, showing that the influence of government transitions on turnover is partly channeled through changes in temporary workforce composition. This suggests that temporary employees are more exposed to turnover risks during political transitions, which in turn amplifies overall turnover. The results underscore the importance of workforce composition, particularly the reliance on temporary contracts, as a key factor in how political shifts affect public sector stability.

1.5.4 Sensitivity analysis.

The sensitivity analysis for the causal mediation model reveals important insights into the robustness of the mediating effect of temporary contract proportions on the relationship between government transitions and turnover. The key metric, ρ, indicates the correlation between the unobserved confounders of the mediator (proportion of temporary contracts) and the outcome (turnover). In this case, $\rho = 0.2$ at the point where the ACME equals zero. This relatively low correlation suggests that only modest levels of unobserved confounding would nullify the mediating effect. Additionally, the R^2_M*R^2_Y* value of 0.04 and R^2_M∼R^2_Y∼ value of 0.0331 further indicate that only a small proportion of the variance in both the mediator and the outcome needs to be explained by unobserved factors for the mediating effect to disappear.

These results imply that the mediating effect of the proportion of temporary contracts on turnover in response to government transitions is sensitive to potential unobserved confounders. However, because the required R^2 values are relatively small, it suggests that while the mediation effect is significant, it could be undermined by even slight levels of unmeasured variables, such as internal agency management practices or organizational culture. This reinforces the importance of considering latent factors when interpreting the mediation effect.

In the context of the influence of government transitions on turnover, the sensitivity analysis highlights that while the mediating role of temporary contracts is important, this relationship may be partially driven by unobserved factors that also affect turnover. Thus, while the results provide evidence for the mediating role of temporary contracts, they also underscore the need for caution in attributing this effect solely to observable variables, as hidden confounders may still play a role.

In summary, the mediation analysis demonstrates that the proportion of temporary contracts serves as a significant mediator in the relationship between government transitions and staff turnover, with approximately 21.4% of the total effect being mediated through this variable. This highlights the critical role of temporary staff in amplifying the turnover effect during political transitions. However, the sensitivity analysis provides a complementary layer by assessing the robustness of this mediation effect. It shows that while the ACME remains significant, the results would become null only under moderate correlations ($\rho = 0.2$) between unobserved confounders affecting both the mediator and the outcome. Therefore, while the mediation effect is robust to some extent, it is susceptible to influence from unobserved factors. Taken together, these analyses affirm that the mediation effect is meaningful but should be interpreted with caution, particularly in the presence of potential unobserved confounders. This combination reinforces the validity of the results while acknowledging the potential limits posed by latent factors.

1.5.5 Propensity Score Matching (PSM) – Mixed-Effects Models and Regression Discontinuity Design (RDD)

Propensity Score Matching (PSM) combined with mixed-effects models offers a robust approach for analyzing the impact of political transitions on turnover in the public sector. PSM controls for observable confounders and reduces selection bias by matching services based on key covariates, such as staffing levels, proportion of temporary contracts, and agency-specific characteristics. This matching process creates a balanced dataset, making it particularly useful when a clear, sharp threshold for treatment—such as in natural experiments—is unavailable. By integrating PSM with mixed-effects models, the analysis captures not only the observable differences between matched services but also accounts for unobserved factors like internal management practices, organizational culture, or historical legacies. These latent factors may independently influence turnover, beyond the immediate political transitions. The use of linear mixed-effects models allows for the partitioning of variance into within-service and between-service components, which helps address service-specific heterogeneity—an advantage that other methods, such as Regression Discontinuity Design (RDD), does not inherently provide.

While RDD is often regarded as a "gold standard" for causal inference because it leverages natural discontinuities (such as election result thresholds) to isolate treatment effects, it has limitations in this context. For RDD to be applied effectively, there must be a well-defined cut-off point, such as a narrow election margin, that separates treated from untreated units. This assumes that services just above and below the threshold are randomly distributed, enabling strong causal interpretation. In theory, RDD offers a more rigorous identification strategy than PSM by controlling for both observed and

unobserved confounders around the threshold. However, in the context of government transitions impacting civil service turnover, there is no such universal cut-off across all services. All services experience some degree of transition, but the effects vary depending on their specific characteristics, which diminishes RDD's applicability.

RDD also focuses on identifying the Local Average Treatment Effect (LATE), meaning the estimated effect is valid only for units near the threshold. This limits the generalizability of the results to the broader population of services, as services near the threshold may not represent the wider state apparatus. Moreover, RDD does not account for service-specific factors or variability, making it unsuitable for capturing the full spectrum of turnover dynamics across heterogeneous state agencies.

In other words, PSM combined with mixed-effects models provides a flexible and generalizable framework. It allows for the estimation of the average treatment effect (ATE) across a wide range of services while accounting for unobserved heterogeneity through random effects. This approach offers an understanding of how political transitions influence turnover across the entire spectrum of public agencies, making it particularly well-suited for analyzing bureaucratic dynamics across a large number of agencies.
